# Distinct RORγt-dependent Th17 immune responses are required for autoimmune pathogenesis and protection against bacterial infection

**DOI:** 10.1016/j.celrep.2024.114951

**Published:** 2024-11-05

**Authors:** Xiancai Zhong, Hongmin Wu, Wencan Zhang, Yun Shi, Yousang Gwack, Hai-hui Xue, Zuoming Sun

**Affiliations:** 1 Department of Immunology & Theranostics, Arthur Riggs Diabetes and Metabolism Research Institute, Beckman Research Institute of the City of Hope, Duarte, CA 91010, USA; 2 Department of Physiology, David Geffen School of Medicine, UCLA, Los Angeles, CA 90095, USA; 3 Center for Discovery and Innovation, Hackensack University Medical Center, Nutley, NJ 07110, USA; 4 Lead contact

## Abstract

T helper (Th)17 cells mediate both protective anti-bacterial immune responses and autoimmune pathogenesis, but the distinct pathways regulating these Th17 responses remain unclear. Retinoid-related orphan receptor γ t (RORγt) is a master transcription factor that governs Th17 cell generation and effector functions. We found that a K256R mutation in RORγt impairs Th17-mediated experimental autoimmune encephalomyelitis (EAE) without affecting the clearance of *Citrobacter rodentium*. This indicates distinct RORγt roles in central nervous system pathogenesis versus gut-associated protective Th17 responses. Mechanically, RORγt/Runx1-dependent upregulation of galectin-3 (Lgals3) and chemokine receptor Ccr6 in CD4^+^ T cells is essential for EAE development but not for bacterial clearance. Moreover, Lgals3 is selectively required for recruiting macrophages to produce interleukin (IL)-1β, which in turn promotes Ccr6 expression on CD4^+^ T cells during EAE pathogenesis. Our findings highlight different RORγt-regulated Th17 pathways in autoimmunity and anti-bacterial immunity, with implications for therapies targeting Th17-mediated autoimmunity while preserving effective anti-bacterial responses.

## In brief

Zhong et al. distinguish the role of RORγt in Th17-mediated autoimmunity from its role in protective anti-bacterial immunity through the differential regulation of *Lgals3* and *Ccr6* expression. These findings highlight the potential for developing RORγt-based therapies that specifically target Th17-mediated autoimmunity without compromising Th17-driven anti-bacterial immune responses.

## Graphical abstract

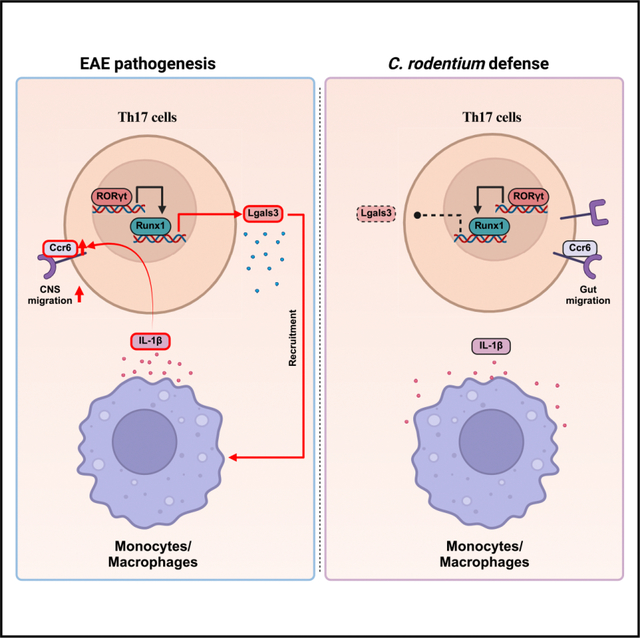

## INTRODUCTION

Interleukin (IL)-17-producing CD4^+^ T cells (T helper [Th]17 cells) orchestrate protective immune responses against bacterial pathogens.^1–5^ However, dysregulated Th17 activity also contributes significantly to the pathogenesis of various autoimmune diseases, such as multiple sclerosis, inflammatory bowel disease, and psoriasis.^6–11^ Thus, there is an urgent clinical need for therapies that can effectively control Th17-mediated autoimmunity.^12,13^ However, treatments that are aimed at mitigating Th17-mediated autoimmunity can also impair Th17-dependent anti-bacterial immune responses. Therefore, understanding the distinct mechanisms behind Th17-mediated autoimmunity versus anti-bacterial responses will be crucial for developing therapeutic strategies that selectively target Th17 dysfunction in autoimmunity while preserving the Th17-mediated anti-bacterial defenses.

The transcription factor retinoid-related orphan receptor γ t (RORγt), encoded by the gene *Rorc*, is critical for Th17 differentiation and effector function.^14–17^ Mutations in *Rorc* significantly impact IL-17 production and lead to severe immune deficiency in both mice and humans.^14,18^ Deletion of the *Rorc* effectively prevents experimental autoimmune encephalomyelitis (EAE), a widely used mouse model for human multiple sclerosis.^14,19–21^ Indeed, numerous pharmacological RORγt inhibitors have been developed for clinical treatment of Th17-dependent autoimmune inflammation.^10,12,13,22^ However, these RORγt inhibitors also suppress Th17-dependent anti-bacterial responses. It remains unknown whether RORγt plays distinct roles in Th17-mediated autoimmunity versus anti-bacterial responses.

In addition to regulating Th17 differentiation, RORγt is also required for T cell development in thymus.^15,16,23,24^ To dissect RORγt function in thymocyte, Th17 differentiation, and effector function of Th17 cells, our previous study mutated all 23 lysines (K) of RORγt to arginines (R) individually,^17^ as K is the potential ubiquitination site, whereas R cannot be ubiquitinated. We found that RORγt-K256R mutation did not affect RORγt function in thymocyte development and Th17 differentiation but impaired effector function of Th17 cells in the induction of pathogenic EAE. Further analysis showed that RORγt-K256R mutation impaired RORγt function in stimulating the expression of *Runx1*, which plays a critical role in Th17-mediated EAE. Additionally, mice expressing RORγt-K256R mutant (*RORγt*^*K256R/K256R*^) had normal thymocyte development and Th17 differentiation but exhibited greatly impaired Th17 autoimmune responses required for EAE development. Surprisingly, we found in this study that *RORγt*^*K256R/K256R*^ mice mounted effective Th17 responses, similar to wild-type (WT) mice, to *Citrobacter rodentium* (*C. rodentium*) infection. Thus, the *RORγt*^*K256R/K256R*^ mouse model presents a unique opportunity to distinguish the roles of RORγt in Th17 function in autoimmunity and anti-bacterial responses. We found that CD4^+^ T cells from the central nervous system (CNS) of EAE-induced *RORγt*^*K256R/K256R*^ mice expressed lower levels of galectin-3 (*Lgals3*) and *Ccr6*. However, forced expression of *Lgals3* or *Ccr6* mostly restored the effector function of *RORγt*^*K256R/K256R*^ CD4^+^ T cells in the induction of EAE. Decreased expression of Lgals3 is due to reduced levels of Runx1, which is required to bind and stimulate the *Lgals3* gene. *Lgals3* promoted the recruitment of IL-1β-producing macrophages, which stimulated *Ccr6* expression in CD4^+^ T cells. In contrast, gut CD4^+^ T cells from *C. rodentium*-infected *RORγt*^*K256R/K256R*^ mice did not show changes in *Lgals3* and *Ccr6* expression. Therefore, our results demonstrate a requirement for RORγt-dependent tissue-specific regulation of *Lgals3* and *Ccr6* expression in Th17-mediated EAE induction that is distinct from the Th17-mediated anti-bacterial responses.

## RESULTS

### *RORγt*^*K256R/K256R*^ mice display impaired Th17-mediated EAE induction

**Figure 1.**
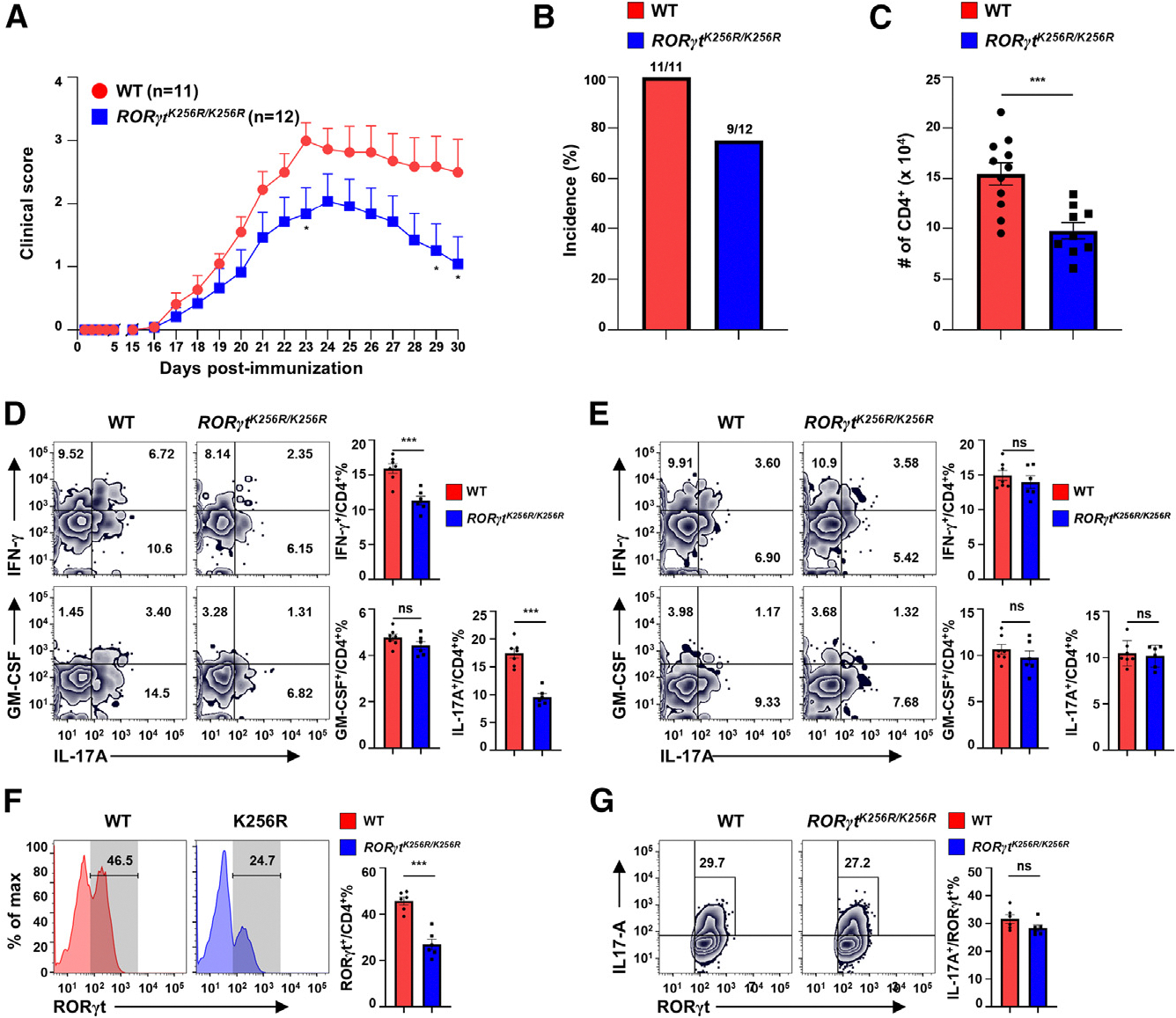
*RORγt*^*K256R/K256R*^ mice display impaired Th17-mediated EAE induction (A) Mean clinical scores at indicated time points for EAE in *Rag1*^−/−^ mice adoptively transferred with 3 × 10^6^ naive CD4^+^ T cells from WT or *RORγt*^*K256R/K256R*^ mice (*n* = 11–12, three independent experiments), followed by immunization with MOG_35–55_. (B) Incidence of EAE in mice shown in A on day 30 post immunization. (C) The number of CD4^+^ T cells infiltrated into the CNS of indicated mice shown in (A). (D and E) Representative flow cytometric analysis (left panels) and percentage (right panels) of IFN-γ^+^/CD4^+^, GM-CSF^+^/CD4^+^, or IL-17A^+^/CD4^+^ cells recovered from CNS (D) or spleen (E) of mice as shown in (A). (F) Representative flow cytometric analysis of RORγt (left panels) and percentage of RORγt^+^ cells among CD4^+^ T cells recovered from CNS of mice shown in (A). (G) Representative flow cytometric analysis of IL-17A/RORγt (left panels) and percentage of IL-17A^+^ cells among RORγt^+^ cells in CD4^+^ T cells recovered from CNS of mice shown in (A). Data are presented as mean ± standard error mean (SEM). Statistical significance is indicated as ***p < 0.001; ns, not significant (two-tailed unpaired Student’s t test). Also see Figure S1.

We previously demonstrated that *RORγt*^*K256R/K256R*^ mice have normal Th17 differentiation but are resistant to Th17-dependent EAE,^17^ suggesting that the Th17 effector function within the context of autoimmune disease was impaired. To specifically determine the function of CD4^+^ T cells, we adoptively transferred naive CD4^+^ T cells from WT or *RORγt*^*K256R/K256R*^ mice to *Rag1*^−/−^-recipient mice for EAE induction. Consistent with the defective Th17 function in *RORγt*^*K256R/K256R*^ mice, the adoptively transferred *RORγt*^*K256R/K256R*^ CD4^+^ T cells induced less severe and fewer incidences of the EAE versus WT donor CD4^+^ T cells (Figures 1A and 1B). Additionally, impaired EAE was associated with reduced *RORγt*^*K256R/K256R*^ CD4^+^ T cell infiltration (Figure 1C), particularly CD4^+^interferon [IFN]-γ^+^ and CD4^+^IL-17A^+^ cells, but not CD4^+^granulocyte-macrophage colony-stimulating factor [GM-CSF]^+^ cells (Figure 1D) in the CNS, indicating a significantly impaired inflammatory response. Furthermore, WT and *RORγt*^*K256R/K256R*^ mice did not show obvious differences in CD4^+^IFN-γ^+^, CD4^+^IL-17A^+^, and CD4^+^GM-CSF^+^ percentages in spleens (Figure 1E), indicating normal Th17 differentiation in the peripheral lymphoid organs. Further analysis revealed a reduction in RORγt^+^ cells (Figure 1F), consistent with the decreased IL-17A^+^ cells (Figure 1D). However, the proportion of IL-17A^+^ cells among RORγt^+^ cells remained unchanged (Figure 1G), in line with our previous finding that RORγt-K256R mutation does not affect Th17 differentiation.^17^ There was no difference in GM-CSF^+^ cells among RORγt^−^ or RORγt^+^ cells between WT and *RORγt*^*K256R/K256R*^ mice (Figure S1A), suggesting that RORγt-K256R does not affect GM-CSF production. Foxp3^+^ Tregs were also equivalent in lymphocytes recovered from the CNS, spleens, and lymph nodes of *Rag1*^−/−^ recipients with WT and *RORγt*^*K256R/K256R*^ cells (Figures S1B–S1D), suggesting that Foxp3^+^ Treg changes did not account for the impaired EAE induction.

### *RORγt*^*K256R/K256R*^ mice mount effective Th17 immune responses against *C. rodentium* infection

**Figure 2.**
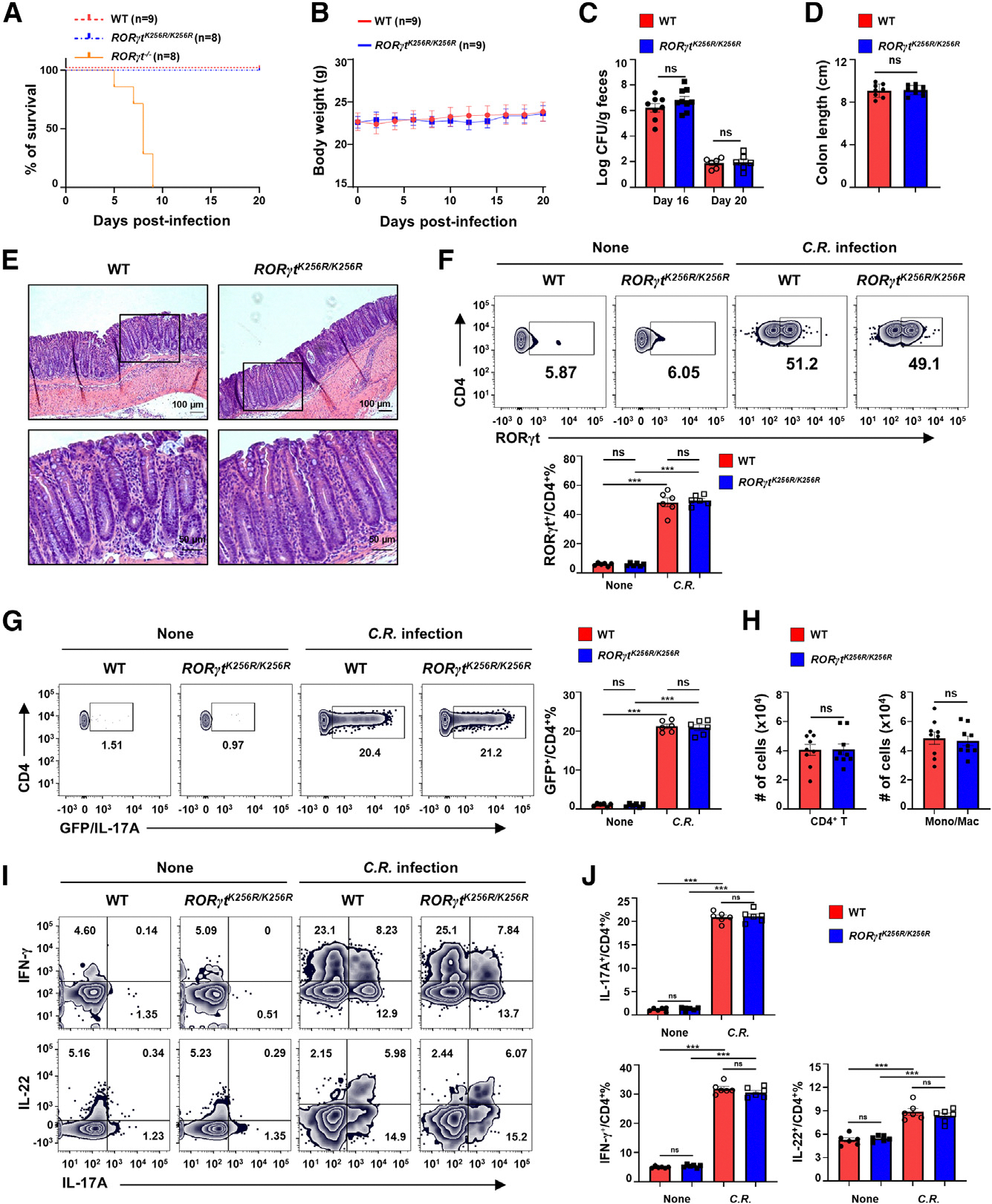
*RORγt*^*K256R/K256R*^ mice mount effective Th17 immune responses against *C. rodentium* infection (A and B) Survival curves (A; *n* = 8–9) and body-weight (B; *n* = 9) of WT, *RORγt*^*K256R/K256R*^, *or RORγt*^−/−^ mice on different days after infection by oral administration of 2 × 10^9^ *C rodentium* (two independent experiments). (C) Bacterial load of indicated mice shown in (A) on day 16 and day 20 post infection (*n* = 6–9). (D and E) Colon length (D; *n* = 8–9) and section of colon (E) of mice shown in (A). (E) Scale bars: 100 mm (top two panels) or 50 μm (bottom two panels). (F) Representative flow cytometric analysis (top panels) and percentage (bottom panel) of RORγt^+^ cells among CD4^+^ T cells in the lamina propria of indicated mice without (none) or with *C. rodentium* (*C.R.*) infection (*n* = 6). (G) Representative flow cytometric analysis (left panels) and percentage (right panels) of IL-17A-GFP^+^ cells among CD4^+^ T cells in the lamina propria of WT/*IL-17A*^*GFP*^ and *RORγt*^*K256R/K256R*^/*IL-17A*^*GFP*^ mice without or with *C. rodentium* infection (*n* = 6). (H) Total number of CD4^+^ T cells (left panel) and monocytes (Mono)/macrophages (Mac, right panel) from the lamina propria of indicated mice shown in (B). (I and J) Representative flow cytometric analysis (I) and percentage (J) of IFN-g^+^, IL-22^+^, or IL-17A^+^ among CD4^+^ T cells recovered from the lamina propria of indicated mice in the absence or presence of *C. rodentium* infection (*n* = 6). Data are presented as mean ± SEM. Statistical significance is indicated as ***p < 0.001; ns, not significant (two-tailed unpaired Student’s t test). Also see Figure S2.

To determine the role of Th17 cells in protection against bacterial infection,^1–3^ we assessed Th17-mediated responses against *C. rodentium*. While all *RORγt*^−/−^ mice eventually succumbed to *C. rodentium* infection due to the lack of Th17 immunity, all WT and *RORγt*^*K256R/K256R*^ mice survived (Figure 2A), maintaining comparable body weight (Figure 2B) and bacterial load (Figure 2C). Bacterial load was high 16 days post infection, but, by 20 days post infection, both WT and *RORγt*^*K256R/K256R*^ mice had much lower bacterial load, suggesting clearance of the infection (Figure 2C). Innate lymphoid cells 3 (ILC3s) mediate the early phase, whereas Th17 cells drive the later adaptive phase of immune responses against *C. rodentium* infection. To rule out the potential influence of ILC3s, we adoptively transferred WT or *RORγt*^*K256R/K256R*^ naive CD4^+^ T cells to *Rag1*^−/−^ mice that normally develop ILC3s. *Rag1*^−/−^ mice without adoptive transferred cells succumbed significantly later (around 20 days post infection; Figure S2A) compared to *RORγt*^−/−^ mice lacking ILC3s (6 days post infection; Figure 2A). Notably, *Rag1*^−/−^ mice that received either WT or *RORγt*^*K256R/K256R*^ CD4^+^ T cells all survived and exhibited similarly low bacterial loads 20–25 days post infection (Figure S2B), supporting effective Th17 responses against infection. *C. rodentium* infection typically leads to colitis as shown by shortened colon length.^25,26^ WT and *RORγt*^*K256R/K256R*^ mice showed equivalent colon length post infection (Figure 2D). Furthermore, no obvious histological differences were observed in the colon between *RORγt*^*K256R/K256R*^ and WT mice (Figure 2E). These results suggest that *RORγt*^*K256R/K256R*^ mice infected with *C. rodentium* mounted a normal Th17-mediated anti-bacterial immune response. Indeed, in the post-infected colon, we observed a greatly increased RORγt^+^ (Figures 2F and S2C) and IL-17A^+^ cells (Figures 2G and S2D) in both *RORγt*^*K256R/K256R*^ and WT mice, along with equivalent numbers of CD4^+^ T cells and monocytes/macrophages in these mice (Figure 2H), confirming normal induction of Th17 cells. Further, analysis of cytokine production revealed similarly upregulated CD4^+^IL-17A^+^, CD4^+^IFNγ^+^, and CD4^+^IL-22^+^ T cells in the colons of *RORγt*^*K256R/K256R*^ and WT mice after infection (Figures 2I and 2J). Therefore, the RORγt-K256R mutation, which impairs Th17 responses in EAE, does not affect Th17 immune responses against *C. rodentium* infection.

### RORγt-K256R mutation decreases *Ccr6* expression on CD4^+^ T cells critical for EAE development but not clearance of *C. rodentium* infection

**Figure 3.**
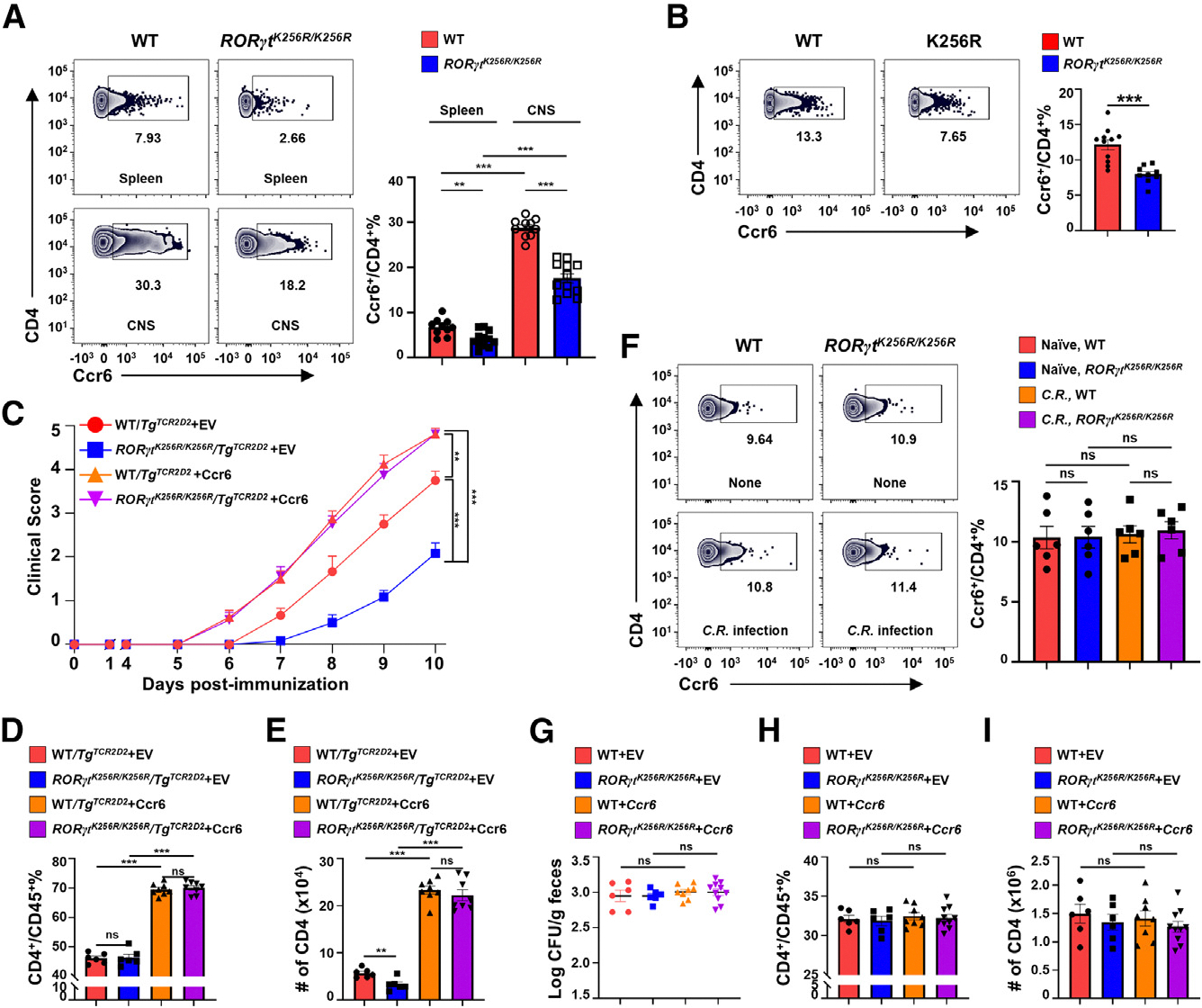
RORγt-K256R mutation decreases the expression of *Ccr6* on CD4^+^ T cells critical for EAE development but not clearance of *C. rodentium* infection (A) Representative flow cytometric analysis (left panels) and percentage (right panel) of Ccr6^+^ among CD4^+^ T cells in the spleen (top) or CNS (bottom) of indicated mice immunized with MOG_35–55_ (*n* = 10–12). (B) Representative flow cytometric analysis (left panels) and percentage (right panel) of Ccr6^+^ among CD4^+^ T cells in the CNS of *Rag1*^−/−^ recipients adoptively transferred indicated naive CD4^+^ T cells and subsequently immunized with MOG_35–55_ as shown in Figure 1A. (C) Mean clinical scores at different day after EAE induction in *Rag1*^−/−^ recipients adoptively transferred with sorted CD4^+^GFP^+^ T cells retrovirally expressing GFP alone (EV) or with Ccr6 and polarized under Th17 conditions (*n* = 6–8, two independent experiments) (D and E) Percentage (D) and number (E) of CD4^+^ T cells among lymphocytes recovered from the CNS of indicated mice shown in (C) at day 10 post immunization. (F) Representative flow cytometric analysis (left panels) and percentage (right panel) of Ccr6^+^ cells among CD4^+^ T cells in the colon of none or *C. rodentium*-infected indicated mice shown in Figure 2B (*n* = 6). (G) Bacterial load in non- or *C. rodentium*-infected *Rag1*^−/−^ recipients adoptively transferred with indicated CD4^+^ T cells retrovirally expressing GFP alone (EV) or together with Ccr6 (*n* = 6–10). (H and I) Percentage (H) and number (I) of CD4^+^ T cells among CD45^+^ lymphocytes in the colon of *Rag1*^−/−^ mice adoptively transferred with indicated cells shown in (G). Data are presented as mean ± SEM. Statistical significance is indicated as **p < 0.05; ***p < 0.001; ns, not significant (two-tailed unpaired Student’s t test). Also see Figure S3.

Defective migration of *RORγt*^*K256R/K256R*^ CD4^+^ T cells into the CNS during EAE development may account for the impaired EAE, as *Ccr6* is required for EAE pathogenesis via regulating CD4^+^ T cell migration into the CNS.^27–29^ We thus monitored Ccr6 expression and found significantly lower Ccr6 levels on CD4^+^ T cells from the CNS and spleens of MOG_35–55_-immunized *RORγt*^*K256R/K256R*^ mice compared to WT mice (Figure 3A). Similarly, CD4^+^ T cell Ccr6 levels were lower in the CNS of EAE-induced *Rag1*^−/−^ recipients adoptively transferred with *RORγt*^*K256R/K256R*^ CD4^+^ T cells (Figure 3B). Notably, the difference in Ccr6 expression was also observed in RORγt^+^ cells (Figure S3A). To determine if low Ccr6 levels led to impaired EAE mediated by *RORγt*^*K256R/K256R*^ CD4^+^ T cells, we utilized 2D2 T cell receptor (TCR) transgenic mice (*Tg*^*TCR2D2*^) that recognize MOG_35–55_.^30,31^ Isolated CD4^+^ T cells from *Tg*^*TCR2D2*^ or *RORγt*^*K256R/K256R*^/*Tg*^*TCR2D2*^ mice were retrovirally transduced with GFP alone (empty virus [EV]) or together with Ccr6 (Figure S3B), followed by *in vitro* Th17 differentiation. Subsequently, GFP^+^CD4^+^ T cells were adoptively transferred in equal number to *Rag1*^−/−^-recipient mice for EAE induction. The expression of Ccr6 in *Tg*^*TCR2D2*^ CD4^+^ T cells significantly enhanced EAE, reinforcing the critical function of Ccr6 in CD4^+^ T cell-mediated EAE (Figure 3C). Compared to the WT CD4^+^ T cells from *Tg*^*TCR2D2*^ mice, CD4^+^ T cells from *RORγt*^*K256R/K256R*^/*Tg*^*TCR2D2*^ mice induced sub-stantially milder EAE in *Rag1*^−/−^ recipients, confirming that the RORγt-K256R mutation impaired Th17 responses in EAE development. Further, forced expression of Ccr6 in *RORγt*^*K256R/K256R*^/*Tg*^*TCR2D2*^ CD4^+^ T cells greatly enhanced their capacity to induce EAE, which was accompanied by a notable increase in the percentage and number of CD4^+^ T cells recovered from the CNS (Figures 3D and 3E). Low Ccr6 levels on *RORγt*^*K256R/K256R*^ CD4^+^ T cells are thus responsible for the impaired EAE. In contrast, CD4^+^ T cells from the colons of WT and *RORγt*^*K256R/K256R*^ mice express equivalent Ccr6 levels regardless of *C. rodentium* infection status (Figure 3F), suggesting that the RORγt-K256R mutation does not affect Ccr6 expression in the colon. Further, forced expression of Ccr6 (Figure S3B) in either WT or *RORγt*^*K256R/K256R*^ CD4^+^ T cells and then adoptively transferred to *Rag1*^−/−^ recipients did not impact the clearance of *C. rodentium* in recipients indicated by similar bacterial loads (Figure 3G), and similar percentage and number of CD4^+^ T cells were recovered from the colon (Figures 3H and 3I). RORγt-K256R mutation thus reduced Ccr6 expression on CD4^+^ T cells and impaired EAE induction. In contrast, the RORγt-K256R mutation does not impact Ccr6 expression on colonic CD4^+^ T cells and does not affect Th17 responses against *C. rodentium* infection.

### RORγt-K256R mutation prevents *Lgals3* (Galectin-3) upregulation to impair EAE induction

**Figure 4.**
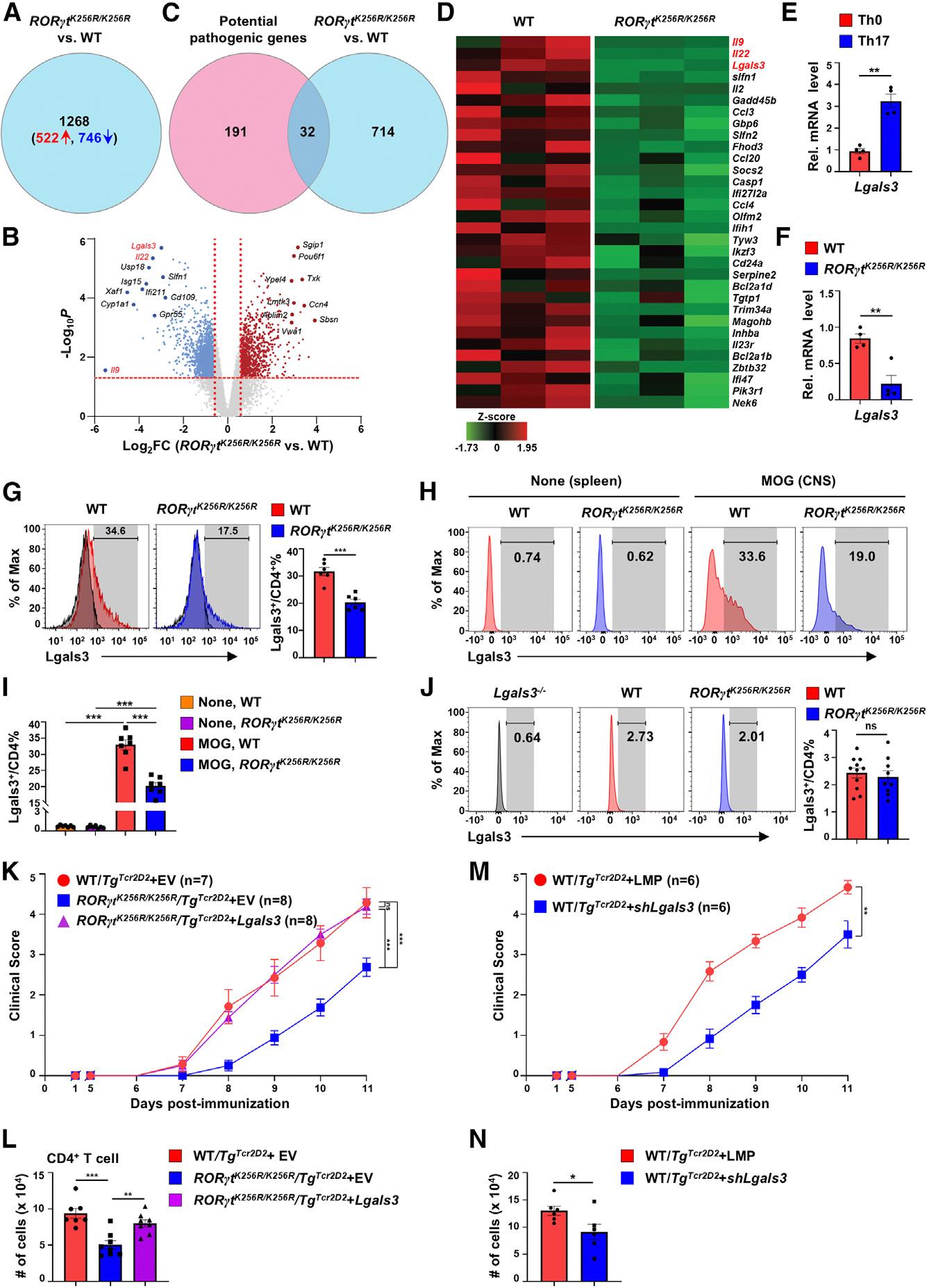
RORγt-K256R mutation prevents *Lgals3* (Galectin-3) upregulation to impair EAE induction (A–E) RNA-seq analysis of WT and *RORγt*^*K256R/K256R*^ CD4^+^ T cells polarized under Th17 conditions. (A) The number of differentially expressed genes (DEGs, black) including upregulated (red) and downregulated (blue) genes with a cutoff at *p* < 0.05 and fold change (FC) >1.9. (B) Volcano plot shows DEGs between indicated Th17 cells (log_10_*p* on y axis and log_2_FC on x axis). Top downregulated (left) and upregulated (right) candidates in *RORγt*^*K256R/K256R*^ cells are indicated. Red font: the top three downregulated candidates. (C) A Venn diagram illustrates the overlapping 32 genes between 223 pathogenic Th17-specific genes and 746 downregulated genes in *RORγt*^*K256R/K256R*^ Th17 cells (GEO: GSE39820).^6,19,33,34^ (D) Heatmap of the 32 overlapping genes described in (C). (E) qPCR analysis of relative mRNA levels of *Lgals3* in Th0 and Th17 cells (*n* = 4). (F and G) qPCR analysis of *Lgals3* mRNA levels (F) and flow cytometric analysis of Lgals3 protein (G, left panels) and percentage (G, right panel) of Lgals3^+^ cells among indicated CD4^+^ T cells polarized under Th17 conditions (*n* = 6). (H and I) Representative flow cytometric analysis (H) and percentage (I) of Lgals3^+^ cells among CD4^+^ T cells from the spleens of indicated untreated mice (left two panels) or from the CNS of indicated EAE-induced mice (right two panels) as described in Figure 1A (*n* = 7). (J) Representative flow cytometric analysis (left three panels) and percentage of Lgals3^+^ cells (right panel) among CD4^+^ T cells in the colons of *C. rodentium*-infected indicated mice described in Figure 2B. *Lgals3*^−/−^ group is a negative staining control. (K) Mean EAE clinical scores at different days of *Rag1*^−/−^ mice adoptively transferred with sorted 1 × 10^5^ indicated CD4^+^GFP^+^ T cells retrovirally expressing GFP alone (EV) or with Ccr6 and polarized under Th17 conditions (*n* = 8, two independent experiments). (L) Number of CD4^+^ T cells recovered from the CNS of EAE-induced mice described in (K). (M) Mean EAE clinical scores of *Rag1*^−/−^-recipient mice adoptively transferred with sorted 1 × 10^5^ *Tg*^*TCR2D*2^ GFP^+^CD4^+^ T cells retrovirally expressing GFP together with scrambled short hairpin RNA (shRNA) or shRNA targeting *Lgals3* (shLgals3) and polarized under Th17 conditions (*n* = 6, two independent experiments). (N) Number of CD4^+^ T cells recovered from the CNS of EAE-induced mice described in (M). Data are presented as mean ± SEM. Statistical significance is indicated as **p < 0.05; ***p < 0.001; ns, not significant (two-tailed unpaired Student’s t test). Also see Figure S4.

To understand how RORγt-K256R affects gene expression responsible for impaired EAE induction, we performed RNA sequencing (RNA-seq) analysis of GFP/IL-17A-expressing WT/*IL-17A-GFP*^*+*^ and *RORγt*^*K256R/K256R*^/*IL-17A-GFP*^*+*^ CD4^+^ T cells differentiated *in vitro* (GEO: GSE211414). A total of 1,268 genes were differentially expressed (*p* < 0.05, fold change [FC] > 1.9) between WT and *RORγt*^*K256R/K256R*^ Th17 cells (Figures 4A and 4B). We further cross-examined these genes with a set of 223 Th17-related genes linked to EAE pathogenesis^6,19,32^ and identified 32 candidates that were specifically upregulated in pathogenic WT Th17 cells but downregulated in *RORγt*^*K256R/K256R*^ Th17 cells (Figures 4C and 4D). *Il9*, *Il22*, and *Lgals3* emerged as the top three candidates (Figures 4B and 4D). Gene Ontology (GO) analysis revealed their involvement in immune function regulation (GO: 0006955 and 0002376; Figure S4A). The expression of these three genes was then further analyzed individually. Although *Il22* mRNA levels were lower in *RORγt*^*K256R/K256R*^ cells (Figures 4D and S4B), IL-22 protein levels showed no obvious difference between WT and *RORγt*^*K256R/K256R*^ Th17 cells (Figure S4C). Notably, IL-22 was almost undetectable in CNS CD4^+^ T cells recovered from MOG_35–55_-immunized mice (Figure S4D). Further, forced expression of IL-22 in *RORγt*^*K256R/K256R*^/*Tg*^*TCR2D2*^ CD4^+^ T cells did not significantly affect EAE development (Figure S4E). Regarding IL-9, although qPCR analysis confirmed lower IL-9 mRNA levels in *RORγt*^*K256R/K256R*^ cells (Figure S4B), very few WT and *RORγt*^*K256R/K256R*^ CD4^+^ T cells expressed this cytokine, both in *in vitro* differentiated Th17 cells (Figure S4F) and in CNS CD4^+^ T cells recovered from EAE-induced mice (Figure S4G). Therefore, IL-22 or IL-9 is unlikely to be a key regulator for RORγt-K256R-affected Th17 function in EAE.

*Lgals3* is a member of the galectin family of carbohydrate-binding proteins and is known to regulate T cell function.^35^ *Lgals3* mRNA is selectively enriched in Th17 versus Th0 cells (Figure 4E, GEO: GSE40918). Compared to differentiated WT Th17 cells, *RORγt*^*K256R/K256R*^ Th17 cells exhibited significantly lower levels of *Lgals3* mRNA (Figure 4F), which was also confirmed via flow cytometry of intracellular Lgals3 protein (Figure 4G). We next monitored Lgals3 levels in EAE-induced mice (Figure 4H). Lgals3 was negligible in CD4^+^ T cells from the spleens of untreated mice (Figure 4H, two left panels, and Figure 4I for quantification). However, Lgals3 was dramatically upregulated in the CNS CD4^+^ T cell infiltrates from both WT and *RORγt*^*K256R/K256R*^ mice immunized with MOG_35–55_ (Figures 4H and 4I), although *RORγt*^*K256R/K256R*^ CD4^+^ T cells failed to upregulate Lgals3 to the levels observed in WT CD4^+^ T cells. Substantial Lgals3 expression was already detected in peripheral CD4^+^ T cells from these immunized mice (Figure S4H), suggesting this upregulation is an early event before entry into the CNS. Upregulation of Lgals3 in activated T cells was reported previously^36^ and also detected in adoptively transferred *Tg*^*TCR2D2*^ CD4^+^ T cells in *Rag1*^−/−^ mice immunized with MOG_35–55_ (Figure S4I). Interestingly, we could not detect upregulation of Lgals3 in colonic CD4^+^ T cells from WT and *RORγt*^*K256R/K256R*^ mice infected by *C. rodentium*, as their levels were equivalent and comparable to *Lgals3*^−/−^ CD4^+^ T cells (Figure 4J). Upregulation of Lgals3 in CNS CD4^+^ T cells from EAE-induced mice but not in colonic CD4^+^ T cells from *C. rodentium*-infected mice indicates a tissue-selective function for Lgals3 in EAE development. To determine the function of *Lgals3* in EAE pathogenesis, *RORγt*^*K256R/K256R*^*/Tg*^*TCR2D2*^ CD4^+^ T cells retrovirally expressed Lgals3 and/or GFP (EV) and subsequently polarized under Th17 conditions *in vitro* (Figure S4J); sorted GFP^+^ cells (Figure S4K) were then adoptively transferred into *Rag1*^−/−^ recipients to induce EAE. Forced *Lgals3* expression did not impact Th17 differentiation in *RORγt*^*K256R/K256R*^*/Tg*^*Tcr2D2*^ CD4^+^ T cells (Figure S4L) yet successfully restored the ability of *RORγt*^*K256R/K256R*^*/Tg*^*Tcr2D2*^ Th17 cells to induce severe EAE (Figure 4K). Additionally, we observed increased infiltration of CD4^+^ T cells in the CNS of *Rag1*^−/−^ recipients reconstituted with *Lgals3*-expressing *RORγt*^*K256R/K256R*^*/Tg*^*Tcr2D2*^ Th17 cells (Figures 4L and S4M). In contrast, knockdown of *Lgals3* (Figure S4N) impaired the induction of Th17-mediated EAE (Figure 4M) and reduced CD4^+^ T cell infiltration into the CNS (Figure 4N). Similar to RORγt-K256R mutation, knockdown of *Lgals3* did not obviously impact Th17 differentiation (Figure S4O), suggesting that both RORγt-K256R mutation and gene silencing of *Lgals3* affect the Th17 effector function only. Altogether, RORγt-K256R mutation controls the pathogenesis Th17-mediated EAE via regulating *Lgals3* expression.

### *Lgals3*^−/−^ mice are defective in pathogenic Th17-mediated EAE but maintain intact protective Th17 immunity against *C. rodentium* infection

**Figure 5.**
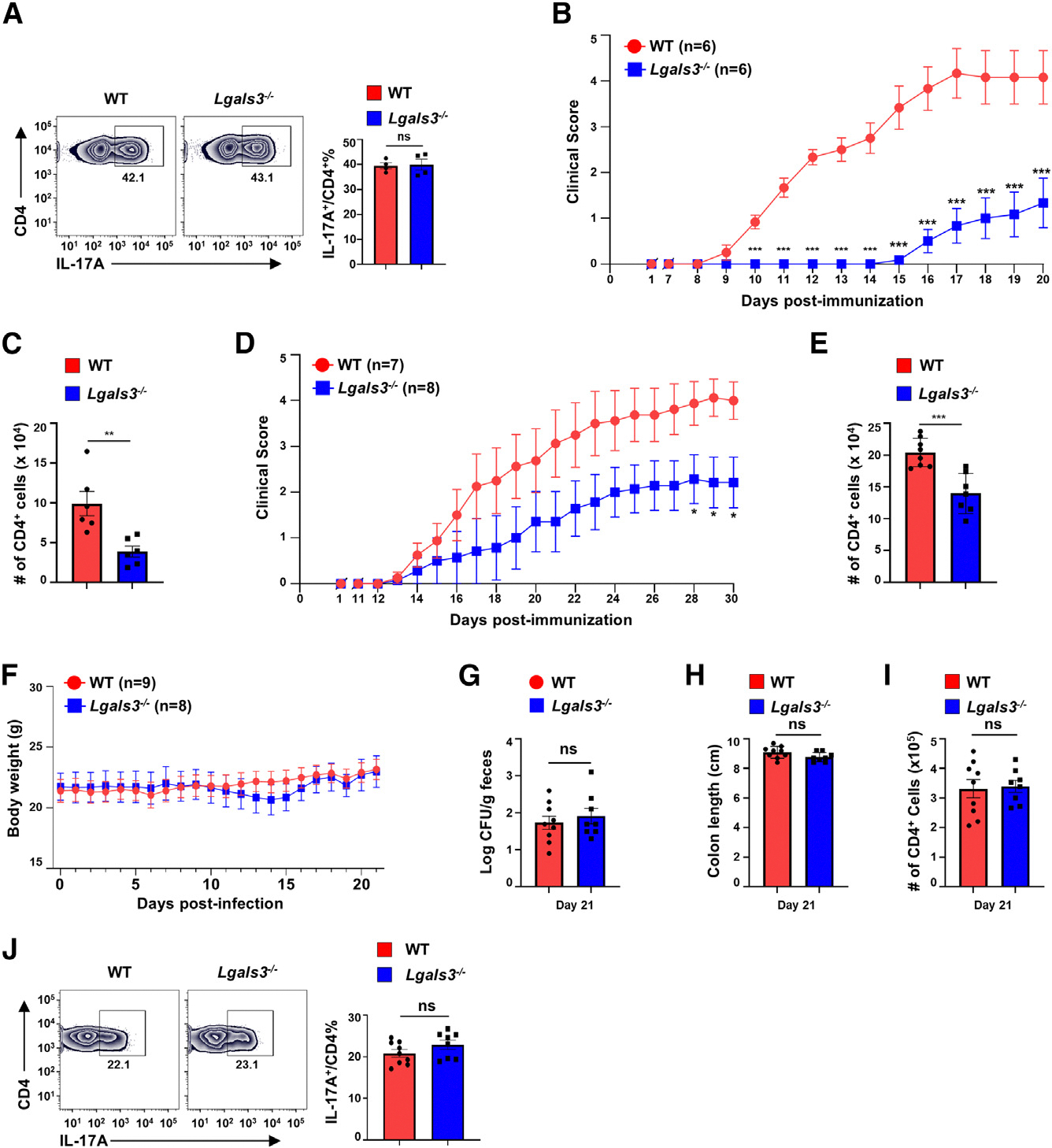
*Lgals3*^−/−^ mice are defective in pathogenic Th17-mediated EAE but maintain intact protective Th17 immunity against *C. rodentium* infection (A) Representative flow cytometric analysis of IL-17A expression in WT and *Lgals3*^−/−^ CD4^+^ T cells polarized under Th17 conditions *in vitro* for 3 days (*n* = 4). (B) Mean clinical score of indicated mice on different days after EAE induction (*n* = 6, two independent experiments). (C) The number of CD4^+^ T cells infiltrated into the CNS of mice described in (B). (D) Mean clinical score of *Rag1*^−/−^ recipients adoptively transferred with 3 × 10^6^ naive CD4^+^ T cells from indicated mice, followed by induction of EAE with MOG_35–55_ (*n* = 7–8, two independent experiments). (E) The number of CD4^+^ T cells infiltrated into the CNS of *Rag1*^−/−^ mice described in (D). (F) Body weight of indicated mice on different days after oral infection with 2 × 10^9^ *C. rodentium* (*n* = 8–9, two independent experiments). (G–I) Bacterial load (G), colon length (H), and the number of CD4^+^ T cells in the colons (I) of mice described in (F) at day 21 post infection. (J) Representative flow cytometric analysis (left panels) and percentage (right panel) of IL-17A^+^ cells among CD4^+^ T cells recovered from the colons of indicated mice shown in (F). Data are presented as mean ± SEM. Statistical significance is indicated as *p < 0.01; **p < 0.05; ***p < 0.001; ns, not significant (two-tailed unpaired Student’s t test). Also see Figure S5.

We next carefully examine *Lgals3* function in Th17 cells using *Lgals3*^−/−^ mice, which were reported to resistant to EAE induction.^37^ CD4^+^ T cells from *Lgals3*^−/−^ mice showed normal upregulation of RORγt (Figure S5A) and IL-17A (Figure 5A) expression upon Th17 polarization, suggesting that Lgals3 was dispensable for Th17 differentiation. *Lgals3*^−/−^ mice exhibited a significantly delayed and impaired EAE induction (Figure 5B) together with decreased CD4^+^ T cell infiltration in the CNS (Figure 5C). Given the germline deletion of *Lgals3* in these mice, we next specifically determined the cell-autonomous function of *Lgals3* in CD4^+^ T cells. For this purpose, naive CD4^+^ T cells were adoptively transferred to *Rag1*^−/−^ mice. *Rag1*^−/−^ recipients with *Lgals3*^−/−^ CD4^+^ T cells developed significantly milder EAE symptoms (Figure 5D), accompanied by reduced CNS CD4^+^ T cell infiltrates (Figure 5E), supporting a cell-autonomous role for *Lgals3* in CD4^+^ T cell-mediated EAE. Regarding Th17 immunity against bacterial infection, *Lgals3*^−/−^ mice exhibited normal clearance of *C. rodentium*, indicated by no obvious differences between WT and *Lgals3*^−/−^ mice in body weight (Figure 5F), bacterial load (Figure 5G), colon length (Figure 5H), and colonic CD4^+^ T cells (Figure 5I), including CD4^+^IL17A^+^ cells (Figures 5J and S5B) and Tregs (Figure S5C). Therefore, *Lgals3* is required for Th17-mediated EAE but is dispensable for Th17 immunity responsible for clearing *C. rodentium* infection.

### RORγt regulates *Lgals3* expression through Runx1

**Figure 6.**
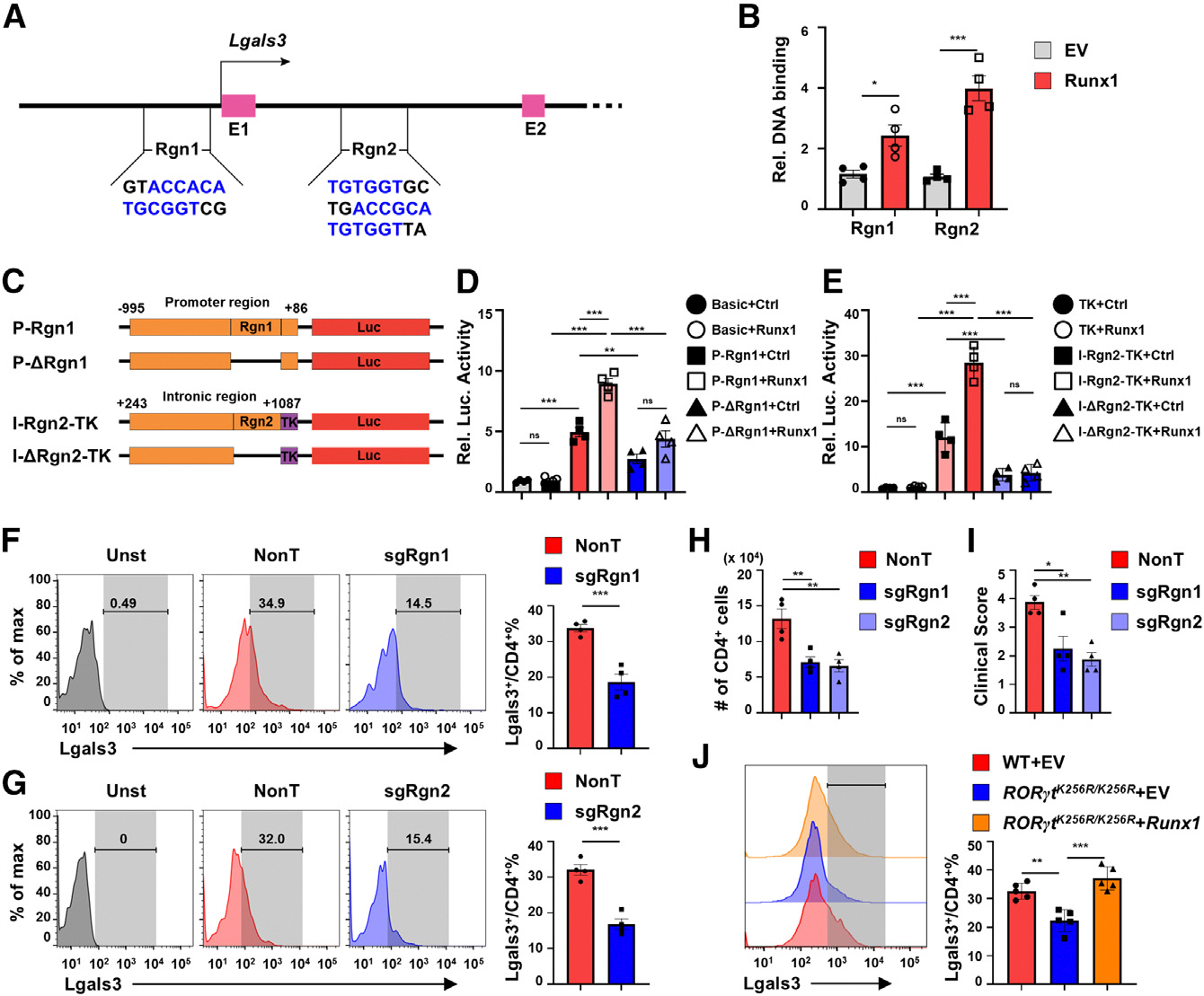
RORγt regulates *Lgals3* expression through the Runx1 transcription factor (A) Scheme of potential Runx1 DNA-binding sites. Region 1 (Rgn1) and Rgn2 are two conserved Runx1-binding sites identified from ChIP-seq for Runx1 (GEO: GSE158093). E, exon. (B) ChIP signals with anti-FLAG antibody in *in vitro* differentiated WT Th17 cells retrovirally transduced with GFP alone (EV) or with FLAG-tagged Runx1 (*n* = 4). (C) Schematic representation of indicated luciferase reporter constructs. P, *Lgals3* promoter; I, *Lgals3* intron; TK, minimal thymidine kinase gene promoter; Δ, deletion; Luc, luciferase. (D and E) Relative luciferase activity from indicated reporter shown in (C) transfected into 293T cells together with expression plasmid for Runx1 or control empty plasmid (Ctrl). Basic is a promoterless reporter (*n* = 4). (F and G) Flow cytometric analysis of Lgal3 expression (left panels) and percentage of Lgals3^+^ cells among Cas9-expressing CD4^+^ T cells retrovirally transduced with nontargeting (NonT) sgRNA controls or sgRNA targeting to delete Rgn1 (F) or Rgn2 (G) and polarized under Th17 conditions (*n* = 4). Gray: controls unstained (Unst) with the Lgals3 antibody. (H) Number of CD4^+^ T cells infiltrating the CNS of *Rag1*^−/−^ adoptively transferred with *Tg*^*Tcr2D*2^ Th17 cells retrovirally transduced with NonT, sgRgn1, or sgRgn2 followed by induction of EAE with MOG_35–55_ (*n* = 5). (I) The endpoint clinical score of mice as described in (H). (J) Flow cytometric analysis of Lgals3 in indicated CD4^+^ T cells expressing GFP alone or together with Runx1. Data are presented as mean ± SEM. Statistical significance is indicated as *p < 0.01; **p < 0.05; ***p < 0.001; ns, not significant (two-tailed unpaired Student’s t test). Also see Figure S6.

To investigate how RORγt regulates *Lgals3* expression, we analyzed chromosome immunoprecipitation sequencing (ChIP-seq) data to determine whether RORγt directly binds to the *Lgals3* locus. However, no significant DNA-binding signals were detected for either RORγt or RORγt^K256R^ at the *Lgals3* locus (Figure S6A), indicating that RORγt does not directly regulate *Lgals3* expression. We next searched the *Lgals3* promoter region for transcription factor-binding sites using the TFBIND online tool (https://tfbind.hgc.jp/) and identified Runx1-binding motifs (Figure 6A). Previously, we have shown that RORγt stimulates *Runx1* expression to regulate the Th17 functions.^17^ RORγt may thus regulate *Lgals3* expression via Runx1. By analyzing public Runx1 ChIP-seq data, we identified two Runx1-binding sites within the *Lgals3* locus: region 1 (Rgn1) in the promoter and region 2 (Rgn2) in the intron (Figures 6A and S6B). ChIP analysis confirmed Runx1 binding at both sites (Figure 6B). To determine the function of Runx1-binding sites, we designed four luciferase reporters: two reporters are driven by a 1-kb *Lgals3* promoter with (P-Rgn1) or without Rgn1 (P-ΔRgn1), and the other two reporters are driven by a basic thymidine kinase (TK) promoter linked to *Lgals3* intron with (I-Rgn2-TK) or without Rgn2 (I-ΔRgn2-TK) (Figure 6C). Indeed, Runx1 stimulated both P-Rgn1 (Figure 6D) and I-Rgn2-TK (Figure 6E) reporter activity, whereas it failed to stimulate either P-ΔRgn1 (Figure 6D) or I-ΔRgn2-TK (Figure 6E), suggesting a role for two Runx1-binding sites in regulating *Lgals3* transcription. To determine the regulation endogenously, we deleted them individually with single guide RNAs (sgRNAs) targeting Rgn1 (sgRgn1) or Rgn2 (sgRgn2) in CD4^+^ T cells from CRISPR-Cas9-expressing mice. Deletion of Rgn1 (Figure 6F) or Rgn2 (Figure 6G) in Th17 polarized cells reduced *Lgals3* expression, suggesting that both Runx1-binding sites positively regulate *Lgals3* expression. The adoptive transfer of *Tg*^*Tcr2D2*^ cells with either sgRgn1 or sgRgn2 to *Rag1*^−/−^ mice led to a significant reduction in CD4^+^ T cell infiltration into the CNS compared to cells transduced with nontargeting (NonT) sgRNA (Figure 6H). Consistently, the deletion of either of the two Runx1-binding sites in CD4^+^ T cells significantly reduced disease severity in the recipient mice (Figure 6I). We have previously shown lower Runx1 levels in *RORγt*^*K256R/K256R*^ Th17 cells.^17^ Forced expression of Runx1 in *RORγt*^*K256R/K256R*^ Th17 cells restored *Lgals3* expression (Figure 6J). Our results suggest that RORγt-K256R mutation downregulates *Runx1*, which is required for binding and stimulating *Lgals3* gene expression.

### *Lgals3* recruits IL-1β-producing macrophages to stimulate CD4^+^ T cell *Ccr6* expression critical for EAE induction

**Figure 7.**
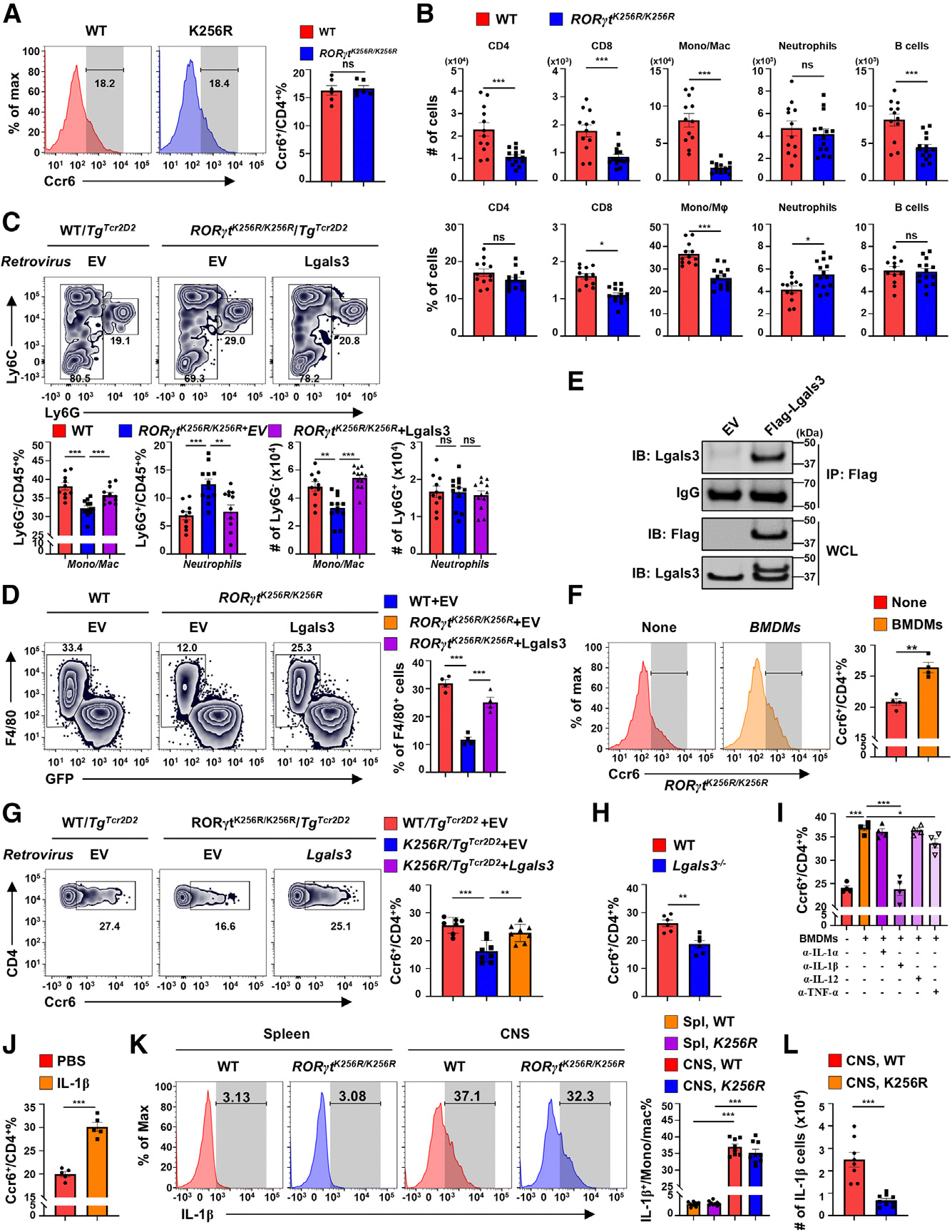
*Lgals3* recruits IL-1β-producing macrophages to stimulate CD4^+^ T cell *Ccr6* expression critical for EAE induction (A) Representative flow cytometric analysis (left panels) and percentage (right panel) of Ccr6^+^ cells among indicated CD4^+^ T cells polarized under Th17 conditions for 3 days (*n* = 6). (B) The number (top panels) and percentage (bottom panels) of indicated types of cells in the CNS of indicated mice immunized with MOG_35–55_. (C) Representative flow cytometric analysis (top panels), percentage (two bottom-left panels), and number (two bottom-right panels) of monocytes/macrophages (Mono/Mac) and neutrophils in the CNS of *Rag1*^−/−^ mice adoptively transferred with indicated CD4^+^GFP^+^ T cells retrovirally expressing GFP alone (EV) or with Lgals3 and polarized under Th17 conditions, followed by EAE induction as described in Figure 4K. (D) Representative flow cytometric analysis (left panels) and percentage (right panel) of BMDMs migrated to the bottom wells containing indicated CD4^+^GFP^+^ T cells retrovirally expressing GFP alone (EV) or with Lgals3 and differentiated under Th17 cells conditions in a Transwell migration assay (*n* = 4 independent experiments). (E) Immunoblot (IB) analysis of FLAG-Lgals3 immunoprecipitated (IP) from the supernatants of CD4^+^ T cells transduced with empty retrovirus (EV) or virus expressing 3xFLAG-Lgals3 and polarized under Th17 condition for 3 days (*n* = 4). Bottom two lanes are the immunoblot analysis of Lgals3 in the whole-cell lysates (WCLs) with anti-FLAG or anti-Lgals3 antibody. (F) Representative flow cytometric analysis (left panels) and percentage (right panel) of Ccr6^+^ cells among *in vitro* differentiated *RORγt*^*K256R/K256R*^ Th17 cells co-cultured without (None) or with BMDMs for 18 h (*n* = 4). (G) Representative flow cytometric analysis (left panels) and percentage (right panel) of Ccr6^+^ cells among CD4^+^ T cells recovered from the CNS of *Rag1*^−/−^ mice adoptively transferred with indicated CD4^+^GFP^+^ T cells retrovirally expressing GFP alone (EV) or with Lgals3 and polarized under Th17 conditions followed by EAE induction as described in Figure 4K (*n* = 7–8). Analysis was conducted on day 10 post immunization. (H) Percentage of Ccr6^+^ cells among CD4^+^ T cells infiltrated into the CNS of WT or *Lgals3*^−/−^ mice immunized with MOG_35–55_ as described in Figure 5B (*n* = 6). (I) Percentage of Ccr6^+^ cells among *in vitro* differentiated Th17 cells co-cultured without (None) or with BMDMs for 18 h in the absence or presence of indicated neutralizing antibodies, analyzed by flow cytometry (*n* = 4). (J) Percentage of Ccr6^+^ cells among *in vitro* differentiated *RORγt*^*K256R/K256R*^ Th17 cells treated with vehicle or recombinant IL-1β (*n* = 5). (K) Representative flow cytometric analysis (left panels) and percentage (right panel) of IL-1β^+^ cells among Mono/Mac cells in the spleens (left two panels) or the CNS (middle two panels) of indicated mice immunized with MOG_35–55_ (n = 8–9). (L) Number of IL-1β^+^ cells among lymphocytes recovered from CNS of indicated EAE-induced mice. Data are presented as mean ± SEM. Statistical significance is indicated as *p < 0.01; **p < 0.05; ***p < 0.001; ns, not significant (two-tailed unpaired Student’s t test). Also see Figure S7.

Our results suggest that RORγt-K256R mutation prevents the upregulation of *Lgals3* and *Ccr6* expression in EAE-involved CD4^+^ T cells, while their expression in CD4^+^ T cells involved in the clearance of *C. rodentium* infection is unaffected. The remaining question is whether there is a link between upregulated Lgals3 and Ccr6 expression observed during EAE induction. Although Ccr6 and Lgals3 expression is downregulated in *RORγt*^*K256R/K256R*^ CD4^+^ T cells *in vivo* from MOG_35–55_-immunized mice (Figures 3A and 4H), we only observed reduced Lgals3 (Figure 4G) but not Ccr6 expression (Figure 7A) in *in vitro* Th17-differentiated *RORγt*^*K256R/K256R*^ CD4^+^ T cells, suggesting that the observed downregulation of Ccr6 is influenced by microenvironment cues *in vivo*. CD4^+^ T cells and monocytes/macrophages (Mono/Mac) were the abundant cell types in the CNS of WT mice that developed EAE (Figure 7B). Interestingly, Lgals3 was reported to regulate the recruitment and activation of macrophages.^38,39^ Coincidentally, significantly lower levels of CNS Mono/Mac were recovered from *RORγt*^*K256R/K256R*^ mice, whereas the total neutrophil infiltration remained the same (Figure 7B). Further, forced Lgals3 expression restored the function of *RORγt*^*K256R/K256R*^*/Tg*^*Tcr2D2*^ CD4^+^ T cells in EAE induction (Figure 4K), which was associated with an increased recovery of Mono/Mac but had no effects on neutrophil from the CNS (Figures 7C and S7A). To determine whether Lgals3 stimulates the recruitment of macrophages, we employed a Transwell migration assay to monitor the migration of macrophages induced by CD4^+^ T cells. *RORγt*^*K256R/K256R*^ CD4^+^ T cells with low Lgals3 expression recruited significantly fewer bone marrow-derived macrophages (BMDMs) compared to WT CD4^+^ T cells (Figures 7D and S7B), whereas the forced Lgals3 expression in *RORγt*^*K256R/K256R*^ CD4^+^ T cells, which was detectable in the culture medium (Figures 7E and S7C), greatly enhanced BMDM recruitment (Figure 7D), suggesting that Lgals3 expression by CD4^+^ T cells promotes the recruitment of BMDMs. Moreover, *in vitro* coculture of differentiated Th17 cells and BMDMs enhanced Ccr6 expression on *RORγt*^*K256R/K256R*^ Th17 cells (Figure 7F), suggesting that BMDMs likely produce secreted factors that stimulate *Ccr6* expression. Further, forced Lgals3 expression in adoptively transferred *RORγt*^*K256R/K256R*^*/Tg*^*TCR2D2*^ Th17 cells into *Rag1*^−/−^-recipient mice restored EAE induction (Figure 4K), which correlated with increased recruitment of Mono/Mac and increased Ccr6 expression levels similar to that observed in WT control cells (Figure 7G). The Ccr6 expression at an earlier stage, day 8 post immunization, also showed restoration in *RORγt*^*K256R/K256R*^*/Tg*^*TCR2D2*^ groups (Figure S7D). In contrast, *Lgals3*^−/−^ mice, which developed impaired EAE (Figure 5B), showed reduced Ccr6 expression on CD4^+^ T cells in the CNS upon EAE induction (Figure 7H). These results suggest that Lgals3 promotes the recruitment of macrophages, which then stimulate the expression of *Ccr6*.

To determine how BMDMs stimulate Ccr6 expression on Th17 cells in BMDM-Th17 coculture experiments (Figure 7F), we used neutralization antibodies against several macrophage-produced cytokines, including IL-1α, IL-1β, IL-12, and tumor necrosis factor (TNF)α. Only IL-1β-neutralizing antibody prevented Ccr6 upregulation on Th17 cells in the presence of BMDMs (Figure 7I), whereas adding recombinant IL-1β to *RORγt*^*K256R/K256R/TgTCR2D2*^ Th17 cells greatly stimulated Ccr6 expression (Figure 7J), suggesting that macrophage-derived IL-1β stimulates Ccr6 expression. Next, we analyzed the expression of IL-1β in Mono/Mac from mice with EAE and found that IL-1β was expressed at low levels in the peripheral Mono/Mac, but it was greatly upregulated in CNS Mono/Mac of EAE-induced mice (Figure 7K). Compared to WT mice, *RORγt*^*K256R/K256R/TgTcr2D2*^ mice only showed consistently slightly decreased percentage of IL-1β^+^ Mono/Mac from the CNS, whereas total number of IL-1β^+^ Mono/Mac cells was greatly reduced (Figure 7L), which led to a substantial reduction in the local IL-1β concentration and thus prevented the upregulation of Ccr6 on *RORγt*^*K256R/K256R*^ CD4^+^ T cells. Thus, Lgals3 stimulates Ccr6 expression, which is critical for Th17 cell-mediated EAE, by promoting the recruitment of IL-1β producing Mono/Mac. Conversely, RORγt-K256R mutation prevents the upregulation of Lgals3 in Th17 cells, which results in a reduced recruitment of IL-1β-producing Mono/Mac. The diminished IL-1β production in the microenvironment, in turn, hinders the upregulation of *Ccr6*, a critical gene for Th17-mediated EAE development.

## DISCUSSION

For the first time, our study distinguished between RORγt-dependent Th17 function in the pathogenesis of autoimmune disease EAE and protection against bacterial infection by *C. rodentium*. Our results support the notion that tissue-specific and context-dependent upregulation of Lgals3 and downstream Ccr6 distinguishes Th17 cell involvement in EAE pathogenesis and clearance of *C. rodentium* infection. Th17 cells responsible for EAE are activated by self-antigen and need to migrate to the CNS by crossing the blood-brain barrier to cause tissue damage. Migration of Th17 cells to the CNS is known to depend on Ccr6.^27–29^ Consistently, we observed greatly enriched CD4^+^Ccr6^+^ T cells in the CNS of EAE-induced mice. The RORγt-K256R mutation prevents the upregulation of Ccr6 on CD4^+^ T cells, which leads to impaired EAE development. On the other hand, Th17 cells are normally present in the gut where abundant bacteria and other microorganisms exist. These gut Th17 cells thus function in maintaining homeostasis and fighting against pathogens such as *C. rodentium*^40^ and are less likely to depend on Ccr6-mediated migration for clearing *C. rodentium* infection. Indeed, we did not observe upregulation of Ccr6 on colonic CD4^+^ T cells in response to *C. rodentium* infection. Further, forced Ccr6 expression enhanced CD4^+^ T cell function in inducing EAE but not clearing *C. rodentium* infection. Therefore, the upregulation of Ccr6 is important for EAE development but not for protection against *C. rodentium* infection.

Our results show that Lgals3 is responsible for upregulating Ccr6 critical for EAE development. Lgals3 has been shown to regulate T cell activation negatively via multiple mechanisms, including altering immunological synapses,^41^ inducing apoptosis,^42^ and inhibiting T cell recruitment.^43^ However, mice with germline knockout of *Lgals3* are resistant to EAE induction, suggesting a positive role for *Lgals3* in the regulation of Th17 cell-mediated immunity.^37^ Indeed, *Lgals3* is identified as a gene specifically expressing in Th17 cells involved in EAE.^6^ We confirm that *Lgals3*^−/−^ mice are defective in Th17 immunity responsible for the pathogenesis of EAE. Furthermore, *Rag1*^−/−^ mice adoptively transferred with *Lgals3*^−/−^ CD4^+^ T cells also displayed defective EAE, demonstrating a cell-autonomous function for *Lgals3* in Th17-mediated EAE. As for Th17 immunity responsible for clearing bacterial infection, our results demonstrate a dispensable role for *Lgals3* in clearing *C. rodentium* infection, which is consistent with a previous observation.^44^ Interestingly, although we observed upregulation of Lgals3 in CD4^+^ T cells during EAE development, Lgals3 was not upregulated in colonic CD4^+^ T cells in response to *C. rodentium* infection, suggesting a tissue-specific function for Lgals3 and supports the selective requirement for Lgals3 in EAE development but not in *C. rodentium* infection clearance.

We demonstrated a link between downregulated Lgals3 and Ccr6 in CD4^+^ T cells responsible for EAE. Ccr6 expression is influenced by the *in vivo* microenvironment.^45^ IL-1β was found to stimulate Ccr6 expression and promote EAE development.^46,47^ Further, Lgals3 recruits IL-1β-producing macrophages upon activation.^38,39,48^ Consistent with this, our results showed that Lgals3 stimulates the recruitment of IL-1β-producing macrophages leading to the upregulation of Ccr6 on migrating CD4^+^ T cells responsible for EAE induction. Our previous report showed that RORγt-K256R mutation impaired effector function of Th17 cells responsible for pathogenic EAE by downregulation of Runx1.^17^ In this study, we showed that Runx1 binds to the *Lgals3* locus to stimulate its expression. Take together, our results demonstrate that RORγt-regulated Runx1 expression promotes the effector function of Th17 cells critical for pathogenic EAE via stimulating Lgals3/Ccr6. In contrast, the Lgals3-Ccr6 pathway is dispensable for Th17-mediated clearance of *C. rodentium* infection. Therefore, tissue-specific disruption of the Lgals3-Ccr6 pathway due to the RORγt-K256R mutation in Th17 cells accounts for the impaired EAE induction phenotype yet allows normal protection against *C. rodentium* infection in the *RORγt*^*K256R/K256R*^ mice.

IL-23 is a pro-inflammatory cytokine that plays a crucial role in the regulation of pathogenic Th17 responses involved in autoimune diseases such as EAE, psoriasis, and inflammatory bowel diseases.^49–51^ Elevated Lgals3 levels are often associated with autoimmunity^52^ and pharmacological inhibition or genetic deletion of *Lgals3* attenuates IL-17-mediated autoimmune diseases such as EAE and colitis.^53^ However, the link between the IL-23 pathway and Lgals3 remains unknown. Our study showed that the IL-23 pathway and *Il23r* are downregulated in *RORγt*^*K256R/K256R*^ CD4^+^ T cells during Th17 differentiation.^17^ It is thus possible that IL-23 regulates Lgals3 expression and activity through its role in promoting chronic inflammation, activating Th17 cells, or recruiting macrophages. However, the specific molecular mechanisms underlying this interaction remain an area of ongoing research, particularly in different disease contexts.

Th17 cells mediate the pathogenic inflammation responsible for many types of autoimmune diseases; targeting Th17 cells is thus a potential treatment for these diseases.^54^ Indeed, inhibiting the Th17 pathway is effective for treating autoimmune conditions, including psoriasis and multiple sclerosis.^55,56^ Considering the essential function of RORγt in Th17 cells, RORγt inhibitors are being developed to treat Th17-dependent autoimmunity.^10,12,13,22,57^ Unfortunately, RORγt inhibitors impact all aspects of Th17 immunity, including those responsible for clearing bacterial infection.^58^ Our results suggest a possible avenue for developing RORγt-based treatments that specifically inhibit autoimmune development while maintaining the protective anti-bacterial Th17 responses.

### Limitations of the study

Our research demonstrated distinct functions for RORγt in Th17-mediated EAE versus protective anti-bacterial immunity. Many of our studies relied on *in vitro* differentiation, which is transient and plastic *in vivo*. *In vivo* real-time monitoring of cellular function is crucial for providing stronger evidence for RORγt in Th17 function. Further, although the K256R mutation prevented ubiquitination at this site, this mutation does not represent the *in vivo* transient nature of most post-translational modifications. Lastly, the enzymes responsible for modifying K256 still remain unknown.

## RESOURCE AVAILABILITY

### Lead contact

Further information and requests for resources and reagents should be directed to and will be fulfilled by the lead contact, Zuoming Sun (zsun@coh.org).

### Materials availability

Plasmids generated in this study will be provided by Dr. Zuoming Sun pending scientific review and a submission of material transfer agreement to mta@coh.org.

### Data and code availability

All data needed to evaluate the paper’s conclusions are present in the paper and/or the supplemental information. The RNA-seq data (GEO: GSE211414) and ChIP-seq data (GEO: GSE211509) from this paper are available in the EO database. This paper does not report original code. Any additional information required to reanalyze the data reported in this paper is available from the lead contact upon request.

## STAR★METHODS

### EXPERIMENTAL MODEL AND STUDY PARTICIPANT DETAILS

#### Mice

All male and female mice used for experiments were between 6 and 12 weeks old; age-matched littermates were used. The *RORγt*^−/−^ and *RORγt*^*K256R/K256R*^ mouse strains were described previously.^17,23^ *Rag1*^−/−^, *Tg*^*TCR2D2*^, *Lgals3*^−/−^, *IL-17A-GFP*, and CRISPR/Cas9-EGFP mice were purchased from the Jackson Laboratory. For some assays, the mice were crossed to generate *RORγt*^*K256R/K256R*^/*Tg*^*TCR2D2*^, and *RORγt*^*K256R/K256R*^/*IL-17A-GFP* mice. All mice were bred into the C57BL/6j background and maintained in a pathogen-free animal facility at City of Hope. All animal experiments were conducted per the protocols approved by the Institutional Animal Care and Use Committee at City of Hope. The sample sizes were chosen based on previous studies of our own and by others in the field.^15^ The sample sizes are indicated in the figure legends or figures. Allocation of mice to experimental groups was random.

#### Induction and assessment of EAE

To investigate the specific function of CD4^+^ T cells in EAE development, we adoptively transferred 3 × 10^6^ naive CD4^+^ T cells (see “Isolation of naive CD4^+^ T cells” section below) from WT, *RORγt*^*K256R/K256R*^, or *Lgals3*^−/−^ mice to *Rag1*^−/−^ recipient mice of the same sex as donors. In certain experiments, we injected 1 × 10^5^ *in vitro* differentiated *Tg*^*Tcr2D2*^ Th17 cells, which were virally transduced (see “retroviral transduction” section below) with empty vector (EV), *Ccr6*, *Lgals3*, or *shLgals3*. The transduced cells positive for GFP expression were sorted using FACSAria Fusion II (BD Biosciences). EAE was induced in the above mentioned mice at day 7 post-adoptive transfer of indicated cells as previously stated.^17^ Specifically, the recipient mice were immunized with two dorsal injections of 100 μg MOG_35–55_ (EK-2110, Hooke Laboratories), followed by an intraperitoneal injection of 120 ng pertussis toxin (PTX) 4 h later, with an additional PTX injection 24 h after the first dose of PTX. For inducing EAE in WT and *Lgals3*^−/−^ mice, a direct immunization and PTX injection was administered. Mice were monitored daily after EAE induction and a clinical score from 0 to 5 was assigned as previously^59^: 0 = no disease; 0.5 = partially limp tail; 1 = paralyzed tail; 2 = hindlimb weakness; 3 = hindlimb paralysis; 4 = hind and fore limb paralysis; 5 = moribundity and death.

#### *C. rodentium* infection

A single colony of *C. rodentium* was cultured overnight in Luria-Bertani (LB) broth without antibiotics at 37°C. The culture was diluted to an optical density at 600 nm (OD_600_) to 0.1 and further grown to the logarithmic phase with OD_600_ to 1. The *C. rodentium* was pelleted and resuspended in PBS after 2 washes. Six-to eight-week-old WT and *RORγt*^*K256R/K256R*^ mice or *Rag1*^−/−^ mice, which were adoptively transferred with *in vitro* differentiated WT and *RORγt*^*K256R/K256R*^ Th17 cells that were retrovirally transduced with EV and *Ccr6*, were subjected to gavage with a 2 × 10^9^ colony-forming unit (CFU) of *C. rodentium* in 200 μL of PBS. The bacterial load in feces from the colons was determined at either day 16 or day 20 post-infection. Additionally, the profile of lymphocytes infiltrated into the lamina propria of colons was assessed at day 20 post-infection.

To assess bacteria load, 2–3 fresh fecal pellets were weighed and then dissolved in PBS to achieve a concentration of 0.1 g/mL. The debris was removed by centrifugation at 400 × *g* for 1 min. The bacteria-containing supernatant was serially diluted and plated on Difco MacConkey agar (BD Biosciences) plates. These plates were incubated overnight at 37°C. The resulting colonies were counted and used to calculate to CFU/g of feces. For the assessment of lymphocyte infiltration in the colonic lamina propria, colon tissues were cut to 1 cm pieces after elimination of feces. These tissue pieces were predigested in RPMI 1640 medium (Corning Inc.) supplemented with 5% FBS, 5 mmol/L EDTA, and 1 mmol/L dithiothreitol (DTT) under gentle rotation (100 rpm) at 37°C using a rotator shaker (New Brunswick) for 20 min. Subsequently, they were vigorously washed three times in RPMI 1640 medium with 2 mM EDTA to remove the epithelium. The remaining lamina propria tissues were passed through a 100-μm strainer and then enzymatically digested in RPMI 1640 medium containing the enzyme mix from the Lamina Propria Dissociation Kit (130–097-410, Miltenyi Biotec) for 30 min at 37°C. Tissues were further dissociated using a gentleMACS Octo dissociator (Miltenyi Biotec) following the m_intestine_01 protocol. Lymphocytes were enriched by Percoll (Cytiva) gradient centrifugation at 2500 rpm at room temperature for 20 min.

#### Adoptive transfer colitis

WT or *RORγt*^*K256R/K256R*^ naive CD4^+^ T cells were isolated from mouse spleens using negative selection (refer to the “Isolation of naive CD4^+^ T cells” section below). A total of 4 × 10^5^ cells were injected intraperitoneally into *Rag1*^−/−^ mice of the same sex as donors. Mice were weighed before T cells transfer and weekly after the injection for up to 8 weeks.

### METHOD DETAILS

#### Plasmids

The MIGR1 vector was kindly provided by Dr. Warren S. Pear (University of Pennsylvania) and was used for retrovirus-mediated gene transduction. MSCV-Runx1 vector was a gift from Dr. Ichiro Taniuchi (RIKEN Center for Integrative Medical Sciences, Japan). MIGR1-RORγt-3xFlag and MIGR1-RORγt^K256R^-3xFlag vectors were generated previously.^17^ The miRNA30-based retroviral LMP vector^60^ was utilized for retrovirus-mediated gene suppression by generating microRNA-30 (miR30)-based short hairpin RNA (shRNA) (Dow et al., 2012). Total RNA from WT Th17 cells (see “in vitro Th17 differentiation” section below) was extracted and subjected to the synthesis of the first strand of cDNA (see “Polymerase chain reaction (PCR)” section below). The coding sequences of murine *Lgals3* (NM_001145953.1) and *Ccr6* (NM_001190333.1) were PCR-amplified from the cDNA sample with restriction sites (Lgals3: XhoI/EcoRI; Ccr6: BglII/XhoI) incorporated into the primers. PCR products were digested with restriction enzymes and then ligated to MIGR1 backbone vector, which had been digested with the corresponding enzymes, generating MIGR1-Lgals3 and MIGR1-Ccr6. For certain experiments, an additional 3xFlag tag sequence (ATGGACTACAAAGACCATGACGGTGATTATAA AGATCATGACATCGATTACAAGGATGACGATGAC) was appended to the 5^’^ end of the *Lgals3* and *Runx1* coding sequence, positioned between BglII and XhoI restriction sites. pGL3-basic and pRL-SV40-Renilla luciferase vectors were purchased from (Promega). pGL3-TK was constructed from the pGL3-basic vector by inserting the core sequence of thymidine kinase (TK) promoter upstream of luciferase coding sequence. pGL3-P-Rgn1, pGL3-P-ΔRgn1, pGL3-I-Rgn2-TK, pGL3-I-ΔRgn2-TK vectors cloned by PCR-amplification of genomic DNA. A MIGR1 backboned vector with dual sgRNA cassettes and one phosphoglycerate kinase (PGK) promoter–TagBFP cassette was previously generated for large gene fragment deletion.^17^

#### Antibodies

The following antibodies were used for flow cytometric analysis unless otherwise specified. Antibodies against CD16/32 (TruStain FcX PLUS, clone 17011E), CD45 (clone 30-F11), CD3 (145–2C11), CD4 (RM4–5), CD8 (53–6.7), CD19 (1D3), Ly6G (1A8), Ccr6 (29–2L17), IFN-γ (XMG-1.2), GM-CSF (MP1–22F9), and IL-9 (RM9A4) were purchased from BioLegend. Antibodies against CD11b (M1/70), Ly6C (HK1.4), IL-17A (eBio17B7), IL-22 (1H8PWSR), IL-10β (NJTEN3), and Foxp3 (FJK-16s) were obtained from eBioscience. Anti-RORγt (Q31–378) was purchased from BD Biosciences. Lgals3 antibodies (M3/38) were obtained from Miltenyi Biotec for flow cytometric analysis or Santa Cruz Biotechnology for the Western blotting. Rabbit anti-hamster immunoglobulin G fraction (55398) from MP Biomedicals and anti-CD3 (145–2C11) and anti-CD28 (37.51) from BioLegend were used for *in vitro* T cell activation. Polyclonal antibodies against IL-1β, IL-1α, TNFα, and IL-12 were purchased from R&D SYSTEMS for neutralization assays.

#### Cell lines

Platinum-E (Plat-E) retroviral packaging cells were purchased from Cell Biolabs and cultured in Dulbecco’s Modified Eagle Medium (DMEM) containing 4.5 g/L glucose, L-glutamine and sodium pyruvate. The culture medium was additionally supplemented with 10% FBS (Corning Inc.), 100 U/mL penicillin, 100 mg/mL streptomycin (Gibco), 1 μg/mL puromycin (Gibco), and 10 μg/mL blasticidin (InvivoGen).

#### Isolation of naive CD4^+^ T cells

Spleens from WT, *RORγt*^*K256R/K256R*^, *Lgals3*^−/−^, WT/*Tg*^*TCR2D2*^, *RORγt*^*K256R/K256R*^/Tg^TCR2D2^, or CRISPR/Cas9-EGFP mice were smashed in a 40-μm cell strainer using RPMI1640 medium (Corning Inc.). Red blood cells were removed using red blood cell lysis buffer (Invitrogen), and the remaining splenocytes were suspended in Robo buffer (STEMCELL Technologies). Single cell suspension was sequentially mixed and incubated with antibody cocktails that recognize non-CD4^+^ T cells, as well as magnetic beads from the Naive CD4^+^ T cellT-cell Isolation Kit (130–104-453, Miltenyi Biotec). The suspension was then passed through LS columns (130–042-401, Miltenyi Biotec) placed in a QuadroMACS separator (130–091-051, Miltenyi Biotec) to negatively select naive CD4^+^ T cells via magnetic cell sorting (MACS). The purity of isolated cells was higher than 95%.

#### *In vitro* Th17 differentiation

Naive CD4^+^ T cells (see “Isolation of naive CD4^+^ T cells” section above) from WT, *RORγt*^*K256R/K256R*^, *Lgals3*^−/−^, WT/*Tg*^*TCR2D2*^, *RORγt*^*K256R/K256R*^*/Tg*^*TCR2D2*^, *RORγt*^−/−^, WT/IL-17A-GFP^+/−^ or *RORγt*^*K256R/K256R*^*/IL-17A-GFP*^*+/−*^ mice were suspended in RPMI-1640 medium (Corning Inc.) supplemented with 10% FBS (Corning Inc.), 2 mmol/L L-glutamine, 100 U/mL penicillin, 100 mg/mL streptomycin, (10378–016, Gibco), and 50 μmol/L β-mercaptoethanol (Gibco), to achieve a concentration of 4 × 10^5^ cells/mL. The cell suspensions were treated with anti-CD3 (0.25 mg/mL; 145–2C11, BioLegend) and anti-CD28 (1 mg/mL; 37.51, BioLegend). Next, 1 mL of cell suspension was gently added to each well of a 24-well plate, which had been precoated with 0.1 mg/mL rabbit anti-hamster immunoglobulin G fraction (55398, MP Biomedicals). Following overnight activation by anti-CD3 and anti-CD28, the cells were subjected to either direct Th17 differentiation in the aforementioned medium with the presence of TGFβ (2 ng/mL; Miltenyi Biotec), IL6 (20 ng/mL; Miltenyi Biotec), anti-IL4 (2 μg/mL; 11B11, BioLegend), and anti–IFN-γ (2 μg/mL; XMG 1.2, BioLegend), or retroviral transduction (see “retroviral transduction” section below) before Th17 differentiation.

#### *In vitro* differentiation of BMDMs

Bone marrow was collected from femurs and tibias of WT mice by flushing with PBS containing 2% FBS. Erythrocytes were lysed by using Red Blood Cell Lysis Buffer (Invitrogen) for 2 min and remaining cells were then washed with Robo buffer (STEMCELL Technology), followed by a filtration with a 40-μm cell strainer. Bone marrow cells containing progenitors were then plated in a Petri dish (Bioland Scientific) in DMEM supplemented with 10% FBS, 20 ng/mL recombinant GM-CSF or 20 ng/mL M-CSF (BioLegend) at 1 × 10^6^ cells/mL. Medium was changed on day 4 to provide fresh cytokines and culture for an additional 3 days. On day 6, BMDMs were treated with 100 ng/mL LPS and IFN-γ to promote inflammatory BMDM. Cells were trypsinized on day 7 and used for experiment.

#### Retroviral transduction

Before generating retrovirus, Plat-E cells were cultured in the aforementioned medium without antibiotics, including puromycin and blasticidin, until they reached 80% confluence. Retroviral vectors, including MIGR1, MIGR1-Lgals3, MIGR1–3xFlag-Lgals3, MIGR1-Ccr6, LMP, LMP-Lgasl3, or vectors for sgRNAs, were transfected to Plat-E cells using BioT transfection reagent (Bioland Scientific). This was followed by a change to fresh medium 24 h later. The virus-containing supernatant was collected at 48 h and 72 h, filtered using a 0.45-μm polyvinylidene difluoride (PVDF) syringe filter (Millipore), and either used to transduce T cells or stored for future use at −80°C. Activated CD4^+^ T cells (see “in vitro Th17 differentiation” section above) had their medium removed and were replaced with filtered viral supernatants containing 10 μg/mL polybrene (Sigma-Aldrich). Subsequently, the cells were incubated in the viral supernatant at 37°C for an additional 3 h, and the culture medium was replaced with fresh culture medium containing polarizing cytokines for *in vitro* Th17 differentiation.

#### CRISPR-Cas9–mediated genomic DNA deletion

A MIGR1-based vector with two cassettes of U6 promoter–driven transcription of single-guide RNAs (sgRNAs) was utilized to achieve the deletion of large fragments encompassing potential Runx1 binding regions in the *Lgals3* gene.^17^ gRNAs were designed using an online tool CRISPOR (http://crispor.tefor.net/), and the sequences are listed in Table S1. The sgRNAs were delivered to Cas9/EGFP-expressing cells via retroviral transduction (see “retroviral transduction” section above). BFP^+^/GFP^+^ CD4^+^ T cells were analyzed for Lgals3 production using the BD LSRFortessa (BD Biosciences).

#### Transwell migration assay

WT or *RORγt*^*K256R/K256R*^ Th17 cells (see “In vitro Th17 differentiation” section above) were suspended in RPMI-1640 medium containing 2% FBS (Corning Inc.), 2 mmol/L L-glutamine, 100 U/mL penicillin, 100 mg/mL streptomycin (10378–016, Gibco), and 50 μmol/L β-mercaptoethanol (Gibco) and then plated into a 24-well plate at 5 × 10^5^ cells/mL/well. BMDMs (see “In vitro differentiation of BMDMs” section above) were trypsinized, and the same number of cells were placed in the upper chamber of a transwell with a 5 μm pore size (Corning Inc.) with the same number. After 18 h of coculture, BMDMs that had penetrated transwell, including cells still attached to the bottom of upper chambers and those already migrated to lower plate wells, were trypsinized. Subsequently, these cells were then combined with floating cells, and 2 × 10^5^ GFP-expressing counting cells were additionally added for flow cytometric analysis. With an equal number of counting cells, the migration ability was presented as the percentage of F4/80^+^ cells among BMDMs and GFP^+^ counting cells by gating out CD4^+^ Th17 cells.

#### RNA-seq and analysis

*In vitro* differentiated naive CD4^+^ T cells from WT/IL-17A-GFP^+/−^ and *RORγt*^*K256R/K256R*^*/IL-17A-GFP*^*+/−*^ mice (see “in vitro Th17 differentiation” section above) were sorted for the IL-17A-expressing cells (GFP^+^CD4^+^) using FACS Aria Fusion II (BD Biosciences). Total RNA from 1 × 10^6^ sorted cells (each group has four replicates from different mice) was extracted using the RNeasy mini kit (QIAGEN) and subjected to quality control, library preparation, and sequencing at Novogene Co. The analysis was performed through Partek Flow. In brief, the sequence reads were aligned to the mouse whole genome (GRCm38) with validation of quality through prealignment and post-alignment QA/QC. Aligned reads were further subjected to quantification using the Partek E/M algorithm and normalization to counts per million (CPM) with 0.001 added to each. The identification of differentially expressed features was performed through the Partek GSA algorithm that applies multiple statistical models to each gene. Genes with total counts over 30 were considered to be expressed in the cells. The expression values of pathogenic genes were extracted and subjected to a heatmap and volcano analysis in R Bioconductor.

#### Chromatin immunoprecipitation (ChIP) and ChIP sequencing (ChIP-Seq)

*In vitro*–activated (see above) WT CD4^+^ T cells that were transduced with retroviruses carrying GFP or 3xFlag-Runx1 were used for ChIP analysis of DNA-binding to Lgals3 gene locus, while *RORγt*^−/−^ CD4^+^ T cells that were retrovirally transduced with GFP, RORγt-3xFlag/GFP, or RORγt^K256R^-3xFlag/GFP were used for CHIP-Seq. After Th17 polarization, 2 × 10^7^ cells were fixed in 1% formaldehyde at room temperature for 10 min to cross-link proteins with chromatin. The reaction was stopped with incubation in glycine for 5 min. Genomic DNA was fragmented with enzyme cocktail (ChIP-IT Express Enzymatic kit, Active Motif) for 10 min as directed. Cell lysates were centrifuged at 15,000 rpm for 10 min to remove debris, and the supernatant was used for immunoprecipitation. An equal amount of DNA was incubated with anti-FLAG (M2, Sigma-Aldrich) overnight, followed by precipitation with protein G agarose beads. Beads complexed with DNA fragments were extensively washed five times, and DNA was eluted, followed by reverse cross-linking. Recovered DNA was either subjected to reverse-transcription quantitative PCR (RT-qPCR) with 1/25 of total amount as DNA template using or NovaSeq with 51–base pair (bp) paired-end sequencing length. Primers used in RT-qPCR are listed in Table S1 and assay was performed using the PowerUp SYBR Green Master Mix (Applied Biosystems) in a QuantStudio 3 Real-Time PCR System (Thermo Fisher Scientific). Gene expression was calculated using 2^—ΔΔCt^ method and normalized to *hemoglobin beta chain complex* (*Hbb*) gene. The values were firstly normalized to the IgG control group and subsequently normalized to EV group. ChIP-Seq reads were analyzed using Partek Flow through alignment to the mm10 mouse genome using the Burrow-Wheeler aligner (BWA). Peaks were identified with the model-based analysis of ChIP-seq 2 (MACS2) tool (version 2.1.1) and quantified with a minimum region size of 50 bp.

#### Flow cytometry

Cells from mice or *in vitro* culture were suspended in Robo buffer (STEMCELL Technologies) and incubated with TruStain FcX (anti-mouse CD16/32) for 10 min to block non-specific binding of immunoglobulin to the Fc receptors. Fluorophore-conjugated antibodies against surface markers were then added to the cell suspensions and incubated for an additional 15 min, followed by the removal of free antibodies. In the case of intracellular proteins to be determined, these cells were further fixed and permeabilized in CytoFix/CytoPerm buffer (BD Biosciences) for determining cytokines or TF Fix/Perm buffer (BD Bioscience) for assessing transcription factors (TFs), at 4°C for 20 min. Next, these cells were washed with Perm/Wash buffer (BD Bioscience) for cytokine detection or TF Perm/Wash buffer (BD Bioscience) for TF assessment. These cells were stained for cytokines in the Perm/Wash buffer or TFs in the TF Perm/Wash buffer at 4°C for 15 min. For cytokine staining, cells were pre-stimulated with phorbol 12-myristate 13-acetate (50 ng/mL, Sigma-Aldrich) and ionomycin (1 mM, Sigma-Aldrich) for 3 h ahead of staining. Meanwhile, GolgiStop (BD Bioscience) was co-treated to block protein transport. To measure IL-10β in monocytes/macrophages, collected cells were incubated in a culture incubator for 4 h with the exclusive inhibition of protein transport using GolgiStop and GolgiPlug (BD Bioscience). Subsequent analysis was performed in the BD LSRFortessa cell analyzer (BD Bioscience) after washing cells.

#### IP and western blotting

For determining the secretion of Lgals3 by Th17 cells, *in vitro* activated WT CD4^+^ T cells were virally transduced with EV, Lgals3, or 3×Flag-Lgals3, which was followed by Th17 differentiation for 3 days (see “in vitro Th17 differentiation” section above). A total 2 × 10^6^ Th17 cells were replated in fresh medium to a 12-well plate and cultured for 18 h. Cells were pelleted by centrifugation at 500 × *g* for 10 min and supernatants from 3 replicates form different mice were pooled for IP of Lagls3 with anti-Lgals3 or anti-Flag. Cells pellets were lysed in radioimmunoprecipitation assay (RIPA) buffer (Sigma-Aldrich) or Pierce IP lysis buffer (Thermo Fisher Scientific). Halt Protease Inhibitor Cocktail (Thermo Fisher Scientific) was freshly added to the buffer just before cell lysis to prevent protein degradation. A total of 20 μg of protein was mixed with NuPAGE LDS Sample Buffer (Thermo Fisher Scientific) and NuPAGE Sample Reducing Agent (Thermo Fisher Scientific) as directed, and then heated at 70°C for 10 min. Subsequently, protein samples were subjected to sodium dodecyl sulfate–polyacrylamide gel electrophoresis (SDS-PAGE) and transferred to the Odyssey Nitrocellulose Membrane (LI-COR Biosciences). After a blocking of the membrane with Intercept Blocking Buffer (LI-COR Biosciences) for 1 h, target proteins were sequentially immunoblotted with relevant primary antibodies and Odyssey fluorescent secondary antibodies (LI-COR Biosciences) followed by measuring fluorescent intensity with LI-COR Odyssey blot imager (LI-COR Biosciences). The eventual samples for western blotting were pooled from 3 different experiments.

#### Luciferase assay

To measure the DNA binding activity, 200 ng of the pGL3-basic, pGL3-P-Rgn1, pGL3-P-ΔRgn1, pGL3-TK, pGL3-I-Rgn2-TK, or pGL3-I-ΔRgn2-TK vector together with the 2 μg of Runx1 vector was delivered to 4 × 10^5^ HEK293T cells seeded in a 6-well plate. Rinella luciferase vector (100 ng) was cotransfected to cells in each group for normalizing different transfection efficiencies. An empty vector was used to adjust the total plasmid DNA to the same amount. Luciferase activity was measured in Dual-Luciferase Reporter Assay System (Promega) per the manufacturer’s instruction at 1-day posttransfection in a Synergy HTX multimode reader (Agilent). To determine luciferase activity, after background subtraction, Firefly luciferase activities were normalized to Renilla luciferase values, followed by an additional normalization of all Firefly to Renilla ratios to the pGL3-basic or pGL3-TK group.

#### Polymerase chain reaction (PCR)

Total RNA was extracted using the RNeasy mini kit (QIAGEN) as directed. Tetro cDNA synthesis kit (Bioline) was used for reverse transcription to synthesize the first strand cDNA. The cDNA samples were subjected to PCR for cloning using Phusion Plus PCR Master Mix (Thermo Fisher Scientific) or quantitative PCR (qPCR) using PowerUp SYBR Green Master Mix (Applied Biosystems) in a QuantStudio 3 Real-Time PCR System (Thermo Fisher Scientific). The primers used for qPCR are listed in Table S1. The amplification efficiency of all primers was tested and optimized. Gene expression was calculated with the 2^−ΔΔCt^ method normalized to the control gene encoding β-actin and Gapdh, and all measurements were performed in triplicate.

### QUANTIFICATION AND STATISTICAL ANALYSIS

Data are presented as mean ± SEM. The results were analyzed for statistical significance with two-tailed unpaired Student’s *t*-test. *p* values are calculated using GraphPad Prism and presented where the statistical significance (*p* < 0.05) was found. ns: not significant.

## Supplementary Material

Supplementary Materials

## Figures and Tables

**KEY RESOURCES TABLE:** 

REAGENT or RESOURCE	SOURCE	IDENTIFIER

Antibodies		
Armenian hamster monoclonal anti-mouse CD3ε	BioLegend	Cat# 100308; RRID:AB_312673
Armenian hamster monoclonal anti-mouse CD3ε	BioLegend	Cat# 100336; RRID:AB_11203705
Armenian hamster monoclonal anti-mouse CD3ε	BioLegend	Cat# 100359; RRID:AB_2616673
Armenian hamster monoclonal anti-mouse CD196 (Ccr6)	BioLegend	Cat# 129816; RRID:AB_2072798
Rat monoclonal anti-mouse CD16/32	BioLegend	Cat# 156604; RRID:AB_2783138
Rat monoclonal anti-mouse CD45	BioLegend	Cat# 103108; RRID:AB_312973
Rat monoclonal anti-mouse CD45	BioLegend	Cat# 103112; RRID:AB_312977
Rat monoclonal anti-mouse CD45	BioLegend	Cat# 103116; RRID:AB_312981
Rat monoclonal anti-mouse CD4	BioLegend	Cat# 100548; RRID:AB_2563054
Rat monoclonal anti-mouse CD8a	BioLegend	Cat# 100706; RRID:AB_312745
Rat monoclonal anti-mouse CD8a	BioLegend	Cat# 100722; RRID:AB_312761
Rat monoclonal anti-mouse CD19	BioLegend	Cat# 152410; RRID:AB_2629839
Rat monoclonal anti-mouse GM-CSF	BioLegend	Cat# 505406; RRID:AB_315382
Rat monoclonal anti-mouse IFN-γ	BioLegend	Cat# 505806; RRID:AB_315400
Rat monoclonal anti-mouse IFN-γ	BioLegend	Cat# 505810; RRID:AB_315404
Rat monoclonal anti-mouse IFN-γ	BioLegend	Cat# 505710; RRID:AB_2832806
Rat monoclonal anti-mouse IL-4	BioLegend	Cat# 504135; RRID:AB_2750404
Rat monoclonal anti-mouse IL-9	BioLegend	Cat# 514103; RRID:AB_2126639
Rat monoclonal anti-mouse Ly6G	BioLegend	Cat# 127618; RRID:AB_1877261
Syrian hamster monoclonal anti-mouse CD28	Biolegend	Cat# 102121; RRID:AB_2810330
Mouse monoclonal anti-mouse RORγt	BD Biosciences	Cat# 562607; RRID:AB_11153137
Rat monoclonal anti-mouse CD11b	Thermo Fisher Scientific	Cat# 12-0112-82; RRID:AB_2734869
Rat monoclonal anti-mouse Ly6C	Thermo Fisher Scientific	Cat# 47-5932-80; RRID:AB_2573991
Rat monoclonal anti-mouse IL-17A	Thermo Fisher Scientific	at# 25-7177-82; RRID:AB_10732356
Rat monoclonal anti-IL-22	Thermo Fisher Scientific	Cat# 12-7221-82; RRID:AB_10597428
Rat monoclonal anti-IL-1β (Pro-form)	Thermo Fisher Scientific	Cat# 12-7114-82; RRID:AB_10732630
Rat monoclonal anti-mouse Galectin-3 (Lgals3)	Miltenyi Biotec	Cat# 130-101-312; RRID:AB_2651794
Rat monoclonal anti-Galectin-3 (Lgals3)	Santa Cruz Biotechnology	Cat# sc-23938; RRID:AB_627658
Mouse monoclonal anti-FLAG^®^	Sigma-Aldrich	Cat# F3165; RRID:AB_259529
Goat polyclonal anti-mouse IL-1α	R&D Systems	Cat# AF-400-NA; RRID:AB_354473
Goat polyclonal anti-mouse IL-1β	R&D Systems	Cat# AF-401-NA; RRID:AB_416684
Goat polyclonal anti-mouse IL-12	R&D Systems	Cat# AF-419-NA; RRID:AB_354485
Goat polyclonal anti-mouse TNF-α	R&D Systems	Cat# AF-410-NA; RRID:AB_354479
Bacterial and virus strains		
*Citrobacter rodentium* DBS100	ATCC	Cat# 51459
Chemicals, peptides, and recombinant proteins		
IL-6	Miltenyi Biotec	Cat# 130-096-685
TGF-β1	Miltenyi Biotec	Cat# 130-095-066
Dulbecco’s Modified Eagle Medium	Corning	Cat# 15-018-CV
RPMI-160 Medium	Corning	Cat# 15-040-CV
Fetal Bovine Serum (FBS)	Corning	Cat# 35-011-CV
Penicillin-Streptomycin-Glutamine	Thermo Fisher Scientific	Cat# 10378016
β-Mercaptoethanol	Thermo Fisher Scientific	Cat# 21985023
Puromycin	Thermo Fisher Scientific	Cat# A1113803
Halt^™^ Protease Inhibitor Cocktail	Thermo Fisher Scientific	Cat# 78443
Pierce^™^ IP Lysis Buffer	Thermo Fisher Scientific	Cat# 87787
Percoll	Cytiva	Cat# 17544501
Phorbol-12-myristate-13 acetate (PMA)	Sigma-Aldrich	Cat# P8139
Ionomycin calcium salt	Sigma-Aldrich	Cat# I0634
Radioimmunoprecipitation assay (RIPA) buffer	Sigma-Aldrich	Cat# R0278
Red Blood Cell Lysing Buffer	Sigma-Aldrich	Cat# R7757
GolgiStop^™^ Protein Transport Inhibitor	BD Biosciences	Cat# BDB554724
Difco^™^ MacConkey agar	BD Biosciences	DF0818-17-3
Critical commercial assays		
Naive CD4+ T cellT-cell Isolation Kit	Miltenyi Biotec	Cat# 130-104-453
Lamina Propria Dissociation Kit	Miltenyi Biotec	Cat# 130-097-410
RNeasy Mini Kit	Qiagen	Cat# 74104
Tetro cDNA synthesis kit	Bioline	Cat# BIO-65043
ChIP-IT Express Enzymatic kit	Active motif	Act# 53009
PowerUp^™^ SYBR^™^ Green Master Mix	Applied Biosystems	Cat# A25742
Phusion^™^ Plus PCR Master Mix	Thermo Fisher Scientific	Cat# F631XL
LIVE/DEAD Fixable Near-IR Dead Cell	Thermo Fisher Scientific	Cat# L34976
BD Pharmingen^™^ Transcription-Factor Buffer Set	Thermo Fisher Scientific	Cat# BDB562574
BD Cytofix/Cytoperm^™^ Fixation/Permeabilization Kit	Thermo Fisher Scientific	Cat# BDB554714
MOG35-55/CFA Emulsion PTX	Hooke Laboratories	Cat# EK-2110
Dual-Luciferase^®^ Reporter Assay System	Promega	Cat# E1910
Deposited data		
RNA-seq data	GEO	GEO: GSE211414
RNA-Seq data	GEO	GEO: GSE40918
ChIP-seq data	GEO	GEO: GSE211509
ChIP-seq data	GEO	GEO: GSE158093
Microarray data	GEO	GEO: GSE39820
Experimental models: Cell lines		
Platinum-E (Plat-E)	Cell Biolabs	RRID:CVCL_B488
Experimental models: Organisms/strains		
*RORγt^K256R/K256R^* mice	This paper	N/A
*RORγt^K256R/K256R^/Tg^TCR2D2^* mice	This paper	N/A
*Rorγt^−/−^* mice	The Jackson Laboratory	RRID:IMSR_JAX:007571
*Rag1^−/−^* mice	The Jackson Laboratory	RRID:IMSR_JAX:002216
*Tg^TCR2D2^* mice	The Jackson Laboratory	RRID:IMSR_JAX:006912
*Lgals3^−/−^* mice	The Jackson Laboratory	RRID:IMSR_JAX:006338
*IL-17A-GFP* mice	The Jackson Laboratory	RRID:IMSR_JAX:018472
CRISPR/Cas9-EGFP	The Jackson Laboratory	RRID:IMSR_JAX:028555
Oligonucleotides		
qPCR primers	N/A	Table S1
gRNA primers	N/A	Table S1
Recombinant DNA		
MIGR1	Dr. Warren S. Pear	RRID:Addgene_27490
MIGR1-RORγt-3xFlag	This paper	N/A
MIGR1-RORγt-K256R-3xFlag	This paper	N/A
MIGR1-Lgals3	This paper	N/A
MIGR1-3xFlag-Lgals3	This paper	N/A
MIGR1-Ccr6	This paper	N/A
MSCV-RunxI	Dr. Ichiro Taniuchi	N/A
PGL3-basic	Addgene	RRID:Addgene_212936
pRL-SV40 Renilla luciferase control	Addgene	RRID:Addgene_27163
PGL3-TK	This paper	N/A
PGL3-P-Rgn1	This paper	N/A
PGL3-P-ΔRgn1	This paper	N/A
PGL3-I-Rgn2-TK	This paper	N/A
PGL3-I-ΔRgn2-TK	This paper	N/A
Software and algorithms		
GraphPad Prism	Graphpad	Version 9.0; RRID:SCR_002798
FlowJo	FlowJo	Version 10.8.1; RRID:SCR_008520

## Highlights

- RORγt-mediated Th17 responses are required for autoimmune EAE and C. *rodentium* clearance
- RORγt-K256R mutation impairs Th17 responses required for EAE but not for clearing C. *rodentium*
- EAE but not clearing C. *rodentium* requires RORγt-stimulated *Lgals3* and *Ccr6* expression
- *Lgals3*^−/−^ mice resist EAE induction but maintain intact Th17 responses against C. *rodentium*
