# Supplementary material for: Distinct RORγt-dependent Th17 immune responses are required for autoimmune pathogenesis and protection against bacterial infection
Source: Cell Rep. Author manuscript; Available in PMC 2025 Mar 24. (PMC11931457; doi:10.1016/j.celrep.2024.114951)
Supplement: Supplementary Materials [file NIHMS2060413-supplement-Supplementary_Materials.pdf]

Cell Reports, Volume *43*

Supplemental information

Distinct RORγt-dependent Th17 immune responses are required for autoimmune pathogenesis and protection against bacterial infection

Xiancai Zhong, Hongmin Wu, Wencan Zhang, Yun Shi, Yousang Gwack, Hai-hui Xue, and Zuoming Sun

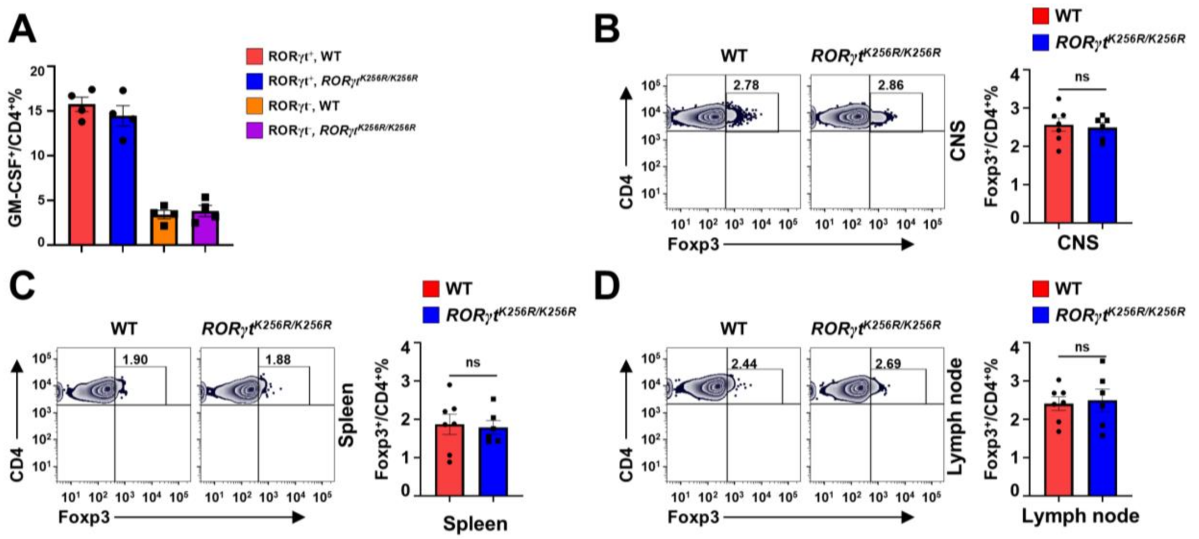

Figure S1. Effect of the RORγt-K256R mutation on GM-CSF production and Tregs, related to Figure 1.

(A) Percentage of GM-CSF^{+} cells among CD4^{+}RORγt^{+} or CD4^{+}RORγt^{-} populations in the CNS of *Rag1*$^{-/-}$ mice adoptively transferred with WT and *RORγt*$^{K256R/K256R}$ naïve CD4^{+} T cells, followed by immunization with MOG$_{35\text{-}55}$ as described in Figure 1A.

(B-D) Representative flow cytometric analysis (left panels) and percentage (right panels) of Foxp3^{+} Tregs in CNS (B), spleens (C) and lymph nodes (D) of *Rag1*$^{-/-}$ mice adoptively transferred with 3 x 10^{6} naïve CD4^{+} T cells from either WT or *RORγt*$^{K256R/K256R}$ mice (n=11-12), followed by immunization with MOG$_{35\text{-}55}$ as described in Figure 1A.

Data are presented as mean ± SEM. ns: not significant (two-tailed unpaired student's *t*-test).

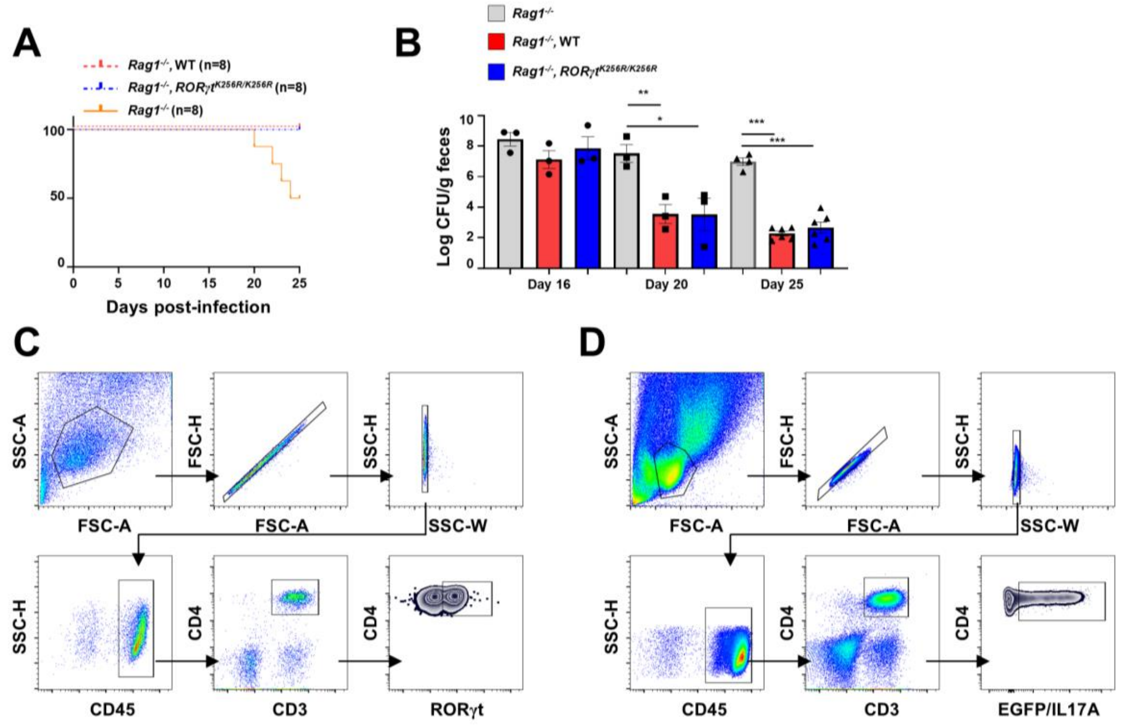

Figure S2. Th17 immune responses against *C. rodentium* infection, related to Figure 2.

(A) Survival curve (n=8) of *Rag1*$^{-/-}$ mice adoptively transferred with or without WT or *RORγt*$^{K256R/K256R}$ naïve CD4^{+} T cells, followed by infection via oral administration of 2 x 10^{9} *C. rodentium*.

(B) Bacterial load of indicated mice shown in A on day 16, day 20, and day 25 post-infection (n=3-6).

(C and D) Gating strategies for the flow cytometric analysis as shown in Figure 2F (C) and 2G (D).

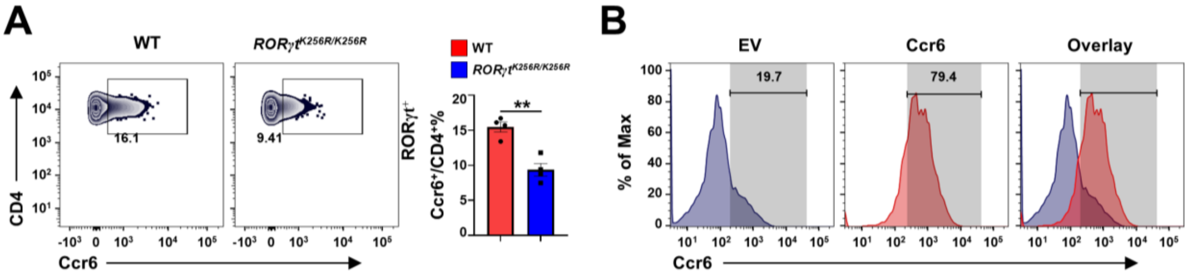

Figure S3. Differential expression of Ccr6 in *RORγt*$^{K256R/K256R}$ CD4$^+$ T cells, related to Figure 3.

(A) Flow cytometric analysis (left panels) and percentage (right panel) of Ccr6$^+$ cells among CD4$^+$RORγt$^+$ cells shown in Figure 3B.

(B) Flow cytometric analysis of Ccr6 expression on *RORγt*$^{K256R/K256R}$/*Tg*^{TCR2D2} CD4$^+$ T cells retrovirally expressing GFP alone (empty virus, EV) or together with Ccr6 for EAE induction after adoptive transfer as described in Figure 3C.

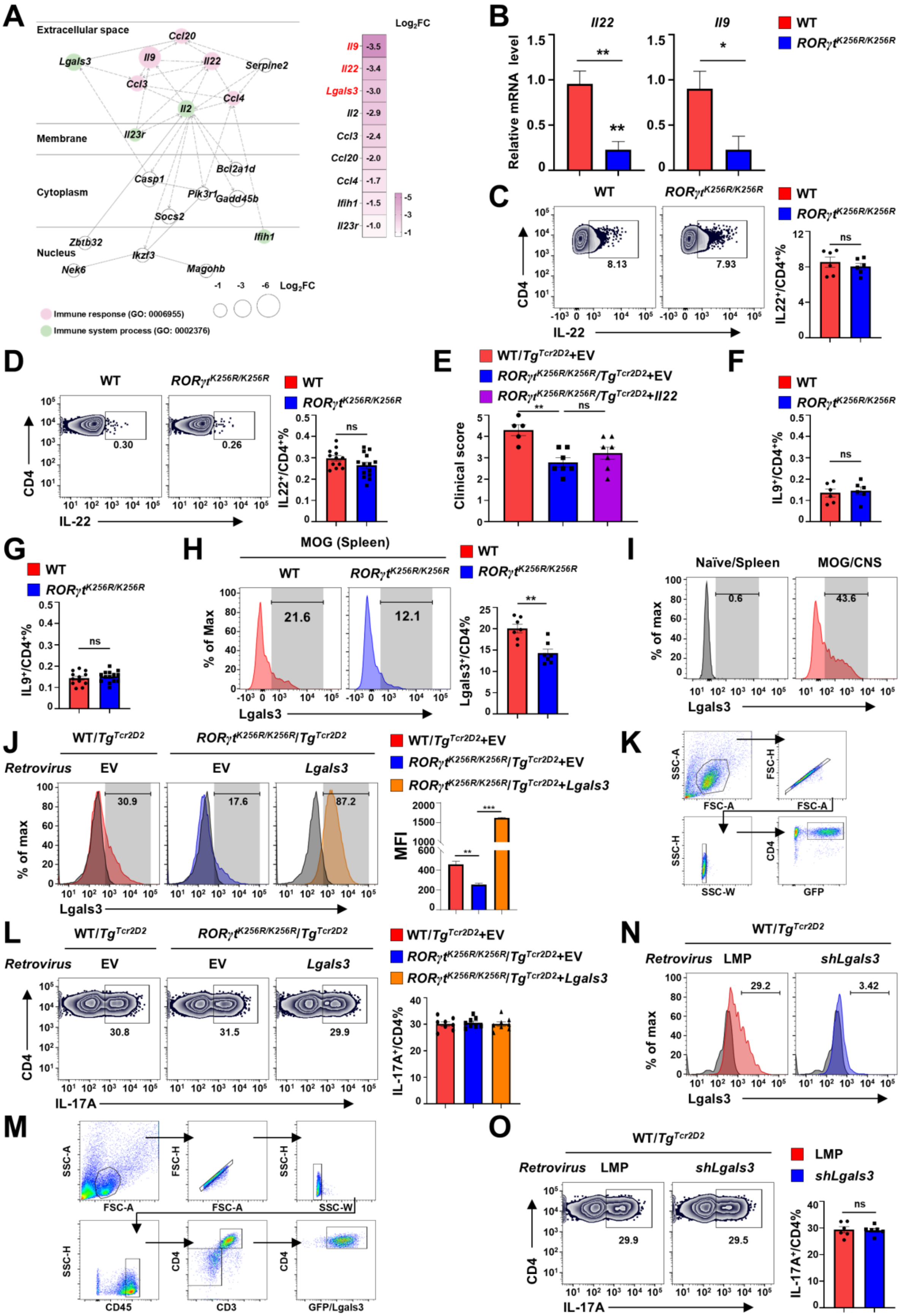
A
Extracellular space
Membrane
Cytoplasm
Nucleus
Ccl20
Lgals3
Il9
Il22
Serpine2
Ccl3
Ccl4
Il2
Il23r
Bcl2a1d
Casp1
Pik3r1
Gadd45b
Socs2
Zbtb32
Ifih1
Ikzf3
Nek6
Magohb
Log2FC
Immune response (GO: 0006955)
Immune system process (GO: 0002376)
Il9 -3.5
Il22 -3.4
Lgals3 -3.0
Il2 -2.9
Ccl3 -2.4
Ccl20 -2.0
Ccl4 -1.7
Ifih1 -1.5
Il23r -1.0
B
Il22
Il9
Relative mRNA level
WT
RORγt K256R/K256R
C
CD4
IL-22
8.13
7.93
IL22+/CD4+%
ns
D
0.30
0.26
E
WT/Tg Tcr2D2+EV
RORγt K256R/K256R/Tg Tcr2D2+EV
RORγt K256R/K256R/Tg Tcr2D2+Il22
Clinical score
F
IL9+/CD4+%
G
H
MOG (Spleen)
% of Max
Lgals3
21.6
12.1
Lgals3+/CD4%
I
Naïve/Spleen
MOG/CNS
% of max
0.6
43.6
J
Retrovirus
EV
Lgals3
30.9
17.6
87.2
MFI
RORγt K256R/K256R/Tg Tcr2D2+Lgals3
K
SSC-A
FSC-A
FSC-H
SSC-H
SSC-W
GFP
L
IL-17A
30.8
31.5
29.9
IL-17A+/CD4%
M
CD45
CD3
GFP/Lgals3
N
WT/Tg Tcr2D2
LMP
shLgals3
29.2
3.42
O
29.9
29.5

Figure S4. Effect of top 3 differentially expressed genes on Th17-mediated EAE pathogenesis.

(A) The overlapping genes in Figure 4C and 4D are subjected to gene interaction network analysis (left) using ingenuity pathway analysis (IPA) software (Qiagen). Nodes are sized proportionally to $\log_2$FC. Gene ontology (GO) analysis is performed using the DAVID database. Genes belonging to immune response and immune system process from GO analysis are colored (green or pink) in the network, and gene expression is shown as a heatmap on the right panel.

(B) qPCR analysis of relative *Il22* and *Il9* mRNA levels in *in vitro* differentiated WT or $ROR\gamma t^{K256R/K256R}$ Th17 cells.

(C and D) Representative flow cytometric analysis (left panels) and percentage (right panel) of IL-22 in *in vitro* differentiated Th17 cells from indicated mice (C) or $CD4^+$ T cells recovered from the CNS of EAE-induced mice with indicated genotype (D).

(E) Mean clinical score of $Rag1^{-/-}$ recipient mice adoptively transferred with *in vitro* Th17 differentiated WT/Tg^{TCR2D2} *or* $ROR\gamma t^{K256R/K256R}$/$Tg^{TCR2D2}$ $CD4^+$ T cells retrovirally expressing GFP alone (empty virus, EV) or together with IL-22. Scores were analyzed at the end of the experiment (n=6).

(F and G) Representative flow cytometric analysis (left panels) and percentage (right panel) of IL-9 in *in vitro* differentiated Th17 cells from indicated mice (F) or $CD4^+$ T cells recovered from the CNS of EAE-induced mice with indicated genotype (G).

(H) Representative flow cytometric analysis of Lgals3 (left panels) and percentage (right panel) of $Lgals3^+$ cells among $CD4^+$ T cells in spleens of indicated mice described in Figure 4H.

(I) Representative flow cytometric analysis of Lgals3 among $CD4^+$ T cells from the spleen of untreated Tg^{TCR2D2} mice (left) or from the CNS of $MOG_{35\text{-}55}$-immunized $Rag1^{-/-}$ mice with adoptive transfer with Tg^{TCR2D2} cells (right).

(J) Representative flow cytometric analysis (left panels) and mean florescence intensity (MFI) of intracellular Lgals3 in *in vitro* Th17 differentiated WT/Tg^{TCR2D2} *or* $ROR\gamma t^{K256R/K256R}$/$Tg^{TCR2D2}$ $CD4^+$ T cells retrovirally expressing GFP alone (EV) or together with Lgals3.

(K and L) Gating strategies for sorting retrovirally transduced GFP^+ cells (K) and flow cytometric analysis of IL-17 expression in indicated $CD4^+$ T cells retrovirally expressing GFP alone or together with Lgals3 and polarized under Th17 cell conditions (L) before adoptive transfer as described in Figure 4K.

(M) Gating strategies for Figure 4L.

(N and O) Representative flow cytometric analysis of Lgals3 (N) and IL-17A (O) in $CD4^+$ T cells transduced with retrovirus expressing scramble shRNA (LMP) or shLgals3 as described in Figure 4M. (N) *Gray*: cells were stained with antibody isotype control.

Data are presented as mean ± SEM. Statistical significance is indicated as *P<0.01; **P<0.05; ***P<0.001; ns: not significant (two-tailed unpaired student's *t*-test).

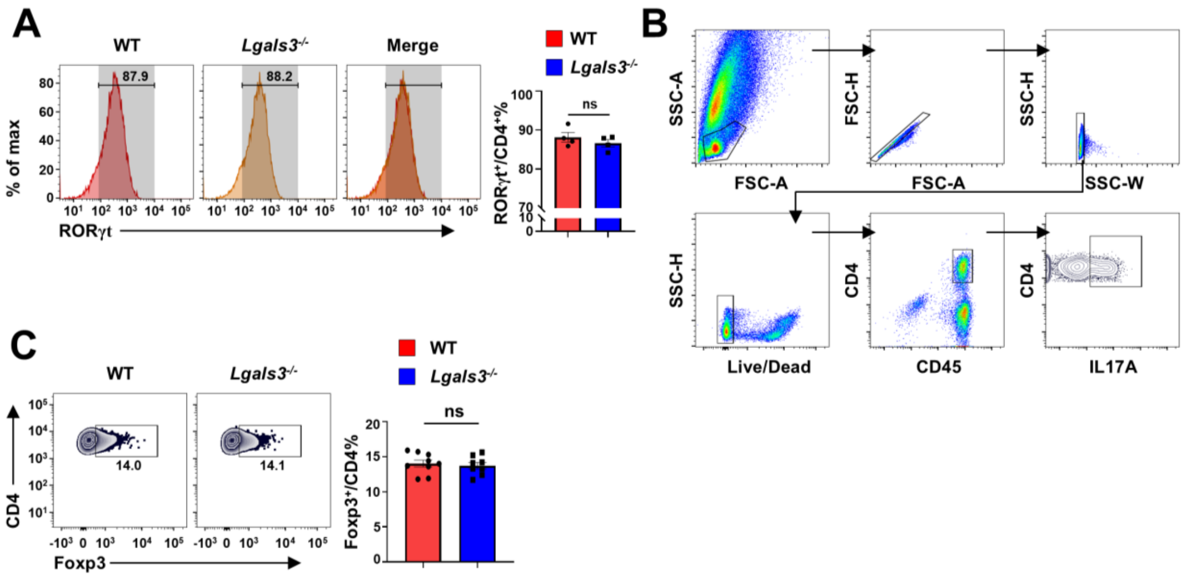

Figure S5. Analysis of Lgals3 on *in vitro* differentiated Th17 and Tregs, related to Figure 5.

(A) Representative flow cytometric analysis (left panels) and percentage (right panel) of RORγt^{+} cells among WT or *Lgals3*$^{-/-}$ CD4^{+} T cells polarized under Th17 conditions for three days.

(B and C) Gating strategies (B) and flow cytometric analysis of Foxp3 expression (C) in colonic CD4^{+} T cells from WT or *Lgals3*$^{-/-}$ mice infected with C. *rodentium* as shown in Figure 5F.

(A and C) Data are presented as mean ± SEM. ns: not significant (two-tailed unpaired student's *t*-test).

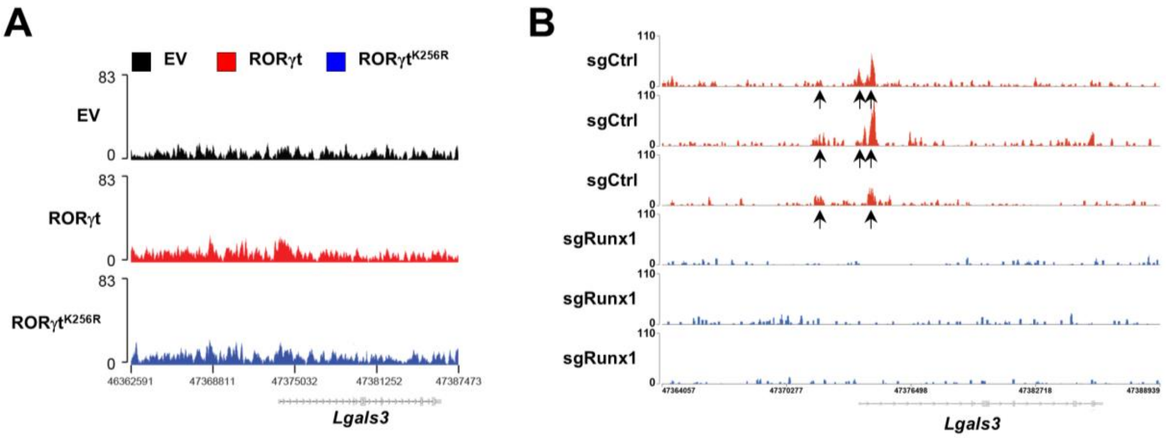

Figure S6. Transcriptional regulation of *Lgals3* gene, related to Figure 6.

(A) ChIP-seq analysis of RORγt DNA-binding signals at the *Lgals3* gene locus in *RORγt*$^{-/-}$ CD4^{+} T cells retrovirally expressing GFP alone (EV), WT RORγt or RORγt^{K256R} mutant (GSE211509) and polarized under Th17 conditions.

(B) ChIP-Seq data showing the binding peaks of Runx1 at the *Lgals3* gene locus in mk4 cells (GSE158093).

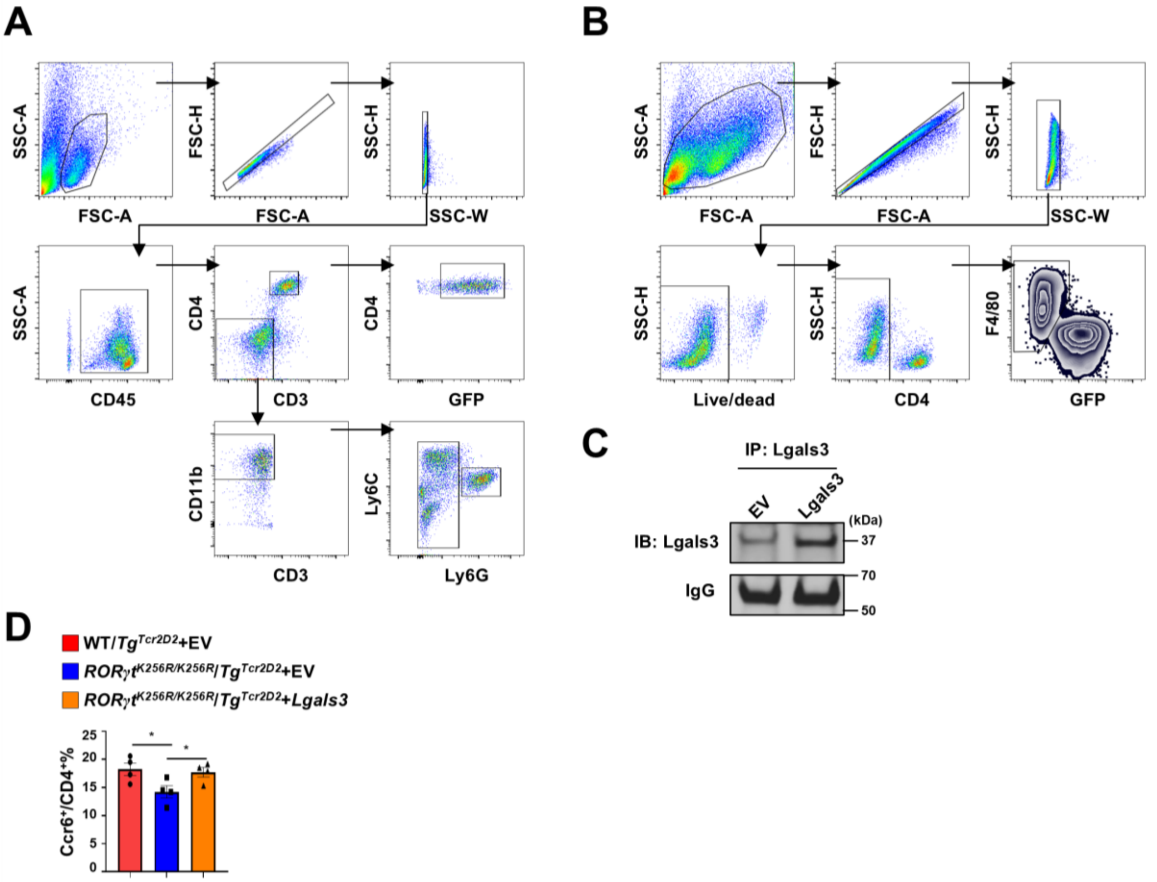

Figure S7. Mechanistic insights into Lgals3 roles in EAE pathogenesis, related to Figure 7.

(A and B) Gating strategies for Figure 7C (A) and 7D (B).

(C) Immunoblot analysis of Lgals3 in CD4$^+$ T cells transduced with retrovirus expressing GFP alone (EV) or with Lgals3 and polarized *in vitro* under Th17 conditions for three days. Equal number (2 x 10^6) of cells are plated to a 12-well plate with fresh medium and additionally cultured for 18 hours.

(D) Percentage of Ccr6$^+$ cells among CD4$^+$ T cells from the CNS of *Rag1*$^{-/-}$ mice, as described in Figure 7G (n=4), on day 8 post-immunization.

Table S1. **List of primer sequences, related to STAR Methods.**

**RT-qPCR**		
**Gene**	**Forward primer**	**Reverse primer**
Lgals3	AGACAGCTTTTCGCTTAACGA	GGGTAGGCACTAGGAGGAGC
Il22	ATGAGTTTTTCCCTTATGGGGAC	GCTGGAAGTTGGACACCTCAA
Il23r	TTCAGATGGGCATGAATGTTTCT	CCAAATCCGAGCTGTTGTTCTAT
Il9	ATGTTGGTGACATACATCCTTGC	TGACGGTGGATCATCCTTCAG
Ccr6	CCTGGGCAACATTATGGTGGT	CAGAACGGTAGGGTGAGGACA
Actb	GGCTGTATTCCCCTCCATCG	CCAGTTGGTAACAATGCCATGT
Gapdh	AGGTCGGTGTGAACGGATTTG	TGTAGACCATGTAGTTGAGGTCA
Lgals3 (Rgn1)	AGGCTTCAGGTGAAGGAATTAG	ACCCAGACTCTCAGACTCAC
Lgals3 (Rgn2)	GTGTCGGGATGAAGAACTACTG	CTCCACCCAGATCTAGAGGTTA
Hbb	GCTCTGGGTACTCCCTCTGA	GCAAATGTGTTGCCAAAAAG
**gRNA**		
NonT #1	AAACTCGCCCGCGTCATAT	ATATGACGCGGGCGAGTTT
NonT #2	AAAGTACCCGCGCGTACGA	TCGTACGCGCGGGTACTTT
Rgn1 #1	TAAAGCCCTAGGCATAGAGT	ACTCTATGCCTAGGGCTTTA
Rgn1 #2	ACTACCCAGGTGAGCGGCGC	GCGCCGCTCACCTGGGTAGT
Rgn2 #1	TTTCTCCGAGAGTTACCCGC	GCGGGTAACTCTCGGAGAAA
Rgn2 #2	GGATTCCGGCTAACCCTAGC	GCTAGGGTTAGCCGGAATCC
